# How Has Intervention Fidelity Been Assessed in Smoking Cessation Interventions? A Systematic Review

**DOI:** 10.1155/2021/6641208

**Published:** 2021-01-15

**Authors:** Suhana Begum, Ayumi Yada, Fabiana Lorencatto

**Affiliations:** ^1^DHealthPsy, City, University of London, London, UK; ^2^City, University of London, London, UK; ^3^University College London, UK

## Abstract

**Introduction:**

Intervention fidelity concerns the degree to which interventions are implemented as intended. Fidelity frameworks propose fidelity is a multidimensional concept relevant at intervention designer, provider, and recipient levels; yet the extent to which it is assessed multidimensionally is unclear. Smoking cessation interventions are complex, including multiple components, often delivered over multiple sessions and/or at scale in clinical practice; this increases susceptibility variation in the fidelity with which they are delivered. This review examined the extent to which five dimensions from the Behaviour Change Consortium fidelity framework (design, training, delivery, receipt, and enactment) were assessed in fidelity assessments of smoking cessation interventions (randomised control trials (RCTs)).

**Methods:**

Five electronic databases were searched using terms “smoking cessation,” “interventions,” “fidelity,” and “randomised control trials.” Eligible studies included RCTs of smoking cessation behavioural interventions, published post 2006 after publication of the framework, reporting assessment of fidelity. The data extraction form was structured around the framework, which specifies a number of items regarding assessment and reporting of each dimension. Data extraction included study characteristics, dimensions assessed, data collection, and analysis strategies. A score per dimension was calculated, indicating its presence.

**Results:**

55 studies were reviewed. There was a wide variability in data collection approaches used to assess fidelity. Fidelity of delivery was the most commonly assessed and linked to the intervention outcomes (73% of the studies). Fidelity of enactment scored the highest according to the framework (average of 92.7%), and fidelity of training scored the lowest (average of 37.1%). Only a quarter of studies linked fidelity data to outcomes (27%).

**Conclusion:**

There is wide variability in methodological and analytical approaches that precludes comparison and synthesis. In order to realise the potential of fidelity investigations to increase scientific confidence in the interpretation of observed trial outcomes, studies should include analyses of the association between fidelity data and outcomes. Findings have highlighted recommendations for improving fidelity evaluations and reporting practices.

## 1. Introduction

Intervention fidelity is defined as the “methodological strategies to monitor and enhance the reliability and validity of behavioural interventions” [1] and the extent to which interventions are implemented as intended. The term fidelity is often used interchangeably with terms such as “quality assurance,” “adherence,” and “treatment integrity” [2]. There has been considerable investment into designing and evaluating the effectiveness of health behaviour change interventions [3], yet comparatively less into investigating how and why these interventions work to achieve intended outcomes. A recent Medical Research Council (MRC) process evaluation guidance [4] has emphasised the importance of investigating fidelity.

Assessing fidelity can inform intervention replication and scalability and is key to promoting research transparency and increasing scientific confidence in interpretation of outcomes [5]. Assessing fidelity can also identify provider training needs and aspects of intervention implementation that could be targeted for improvement in future adaptations [6]. Behaviour change interventions are often complex, comprising multiple components [4]. Behavioural interventions may also involve tailoring to meet the needs of the individuals taking part [7]. Health behaviour change interventions are an example of such complex interventions. In addition to multiple interacting components and tailoring, these interventions are often delivered in busy and unpredictable settings, by diverse intervention providers, to a wide range of recipients [8]. Combined, these factors increase susceptibility to variable fidelity by increasing the opportunities and avenues where intervention variability could potentially be introduced at the designer, provider, and recipient levels.

**Figure 1:**
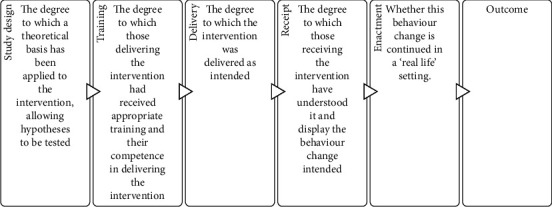
Five dimensions of the BCC framework [1, 6].

Fidelity itself is an equally complex concept [9]. There are numerous models of fidelity from various disciplines, which vary in their conceptualisation and proposed measurement of fidelity [10, 11]. In recognition of this, the National Institutes of Health (NIH) Behaviour Change Consortium (BCC) synthesised numerous fidelity frameworks into an integrated fidelity framework [1, 6]. This framework proposes that fidelity is relevant at the intervention designer, provider, and recipient levels, representing a fidelity pathway to outcomes. It proposes five fidelity dimensions (Figure 1).

This framework recommends strategies for assessing, enhancing, and reporting each dimension. It is important to assess fidelity across all five, as a lack of fidelity to just one could detrimentally impact intervention outcomes. The framework consists of a checklist outlining key fidelity concepts in each of the five dimensions, which (when present in a study) ensure good fidelity. The checklist contains items which are fidelity assessment strategies (e.g., there is a plan for the assessment of whether or not the active ingredients were delivered) as well as fidelity enhancement strategies (e.g., use of treatment manual).

Despite the recognised importance of fidelity and the availability of guidance for assessing and reporting it, fidelity is not frequently investigated for complex interventions. A review of evaluations of intervention programmes published between 1980 and 1994 identified only 24% assessed fidelity [12]. Borrelli et al. [6] reviewed 342 health behaviour change interventions and identified 22% reported strategies for maintaining provider skills (linked to training), 27% reported checking adherence against the protocol (delivery), and 35% reported using treatment manuals to guide the intervention (delivery). 12% of the total studies reported using all three strategies for fidelity whilst 54% reported none. Fidelity assessments do not appear to be improving over time. A more recent review of 28 adult physical activity interventions [5] identified that delivery was the most commonly assessed fidelity domain. Similarly, a review of fidelity assessments in 65 physiotherapist delivered physical activity/exercise interventions identified only 40% of studies reporting on two or more of the fidelity domains [13]. Both reviews noted wide variation in methods used to assess fidelity and that fidelity of design was the least investigated domain.

Other reviews have focused on the assessment of specific fidelity dimensions. Rixon et al. [14] focused on fidelity of “receipt” amongst studies citing the use of the NIH BCC framework. A total of 33 studies were identified, with 19.6% addressing receipt and 12.1% including strategies to enhance receipt. Similarly, Walton et al. [15] reviewed the assessment of fidelity of “delivery” and engagement in 66 health behaviour change interventions, identifying that 32% measured engagement, 30% measured fidelity of delivery, and 36% measured both. They found similar numbers of studies used observational and self-report measures, but noted self-report measures have limitations and observational measures are recommended as the gold-standard measure. They also noted that objective measures such as intervention records were used but these do not measure the participant's understanding of the intervention. Objective measures (e.g., participants demonstrating the skills) were not used by any studies.

Fidelity assessment studies often do not explore association between fidelity and outcomes [6]. As such, there is limited data available on whether extent of fidelity has a positive or detrimental impact on outcomes. Available evidence suggests that greater fidelity is associated with improved outcomes [1]. There is also contrasting views on this matter, as others argue that adaptation and tailoring, which may result in loss of fidelity/standardisation, are important to effecting change.

### 1.1. Fidelity in Smoking Cessation Interventions

Smoking cessation behavioural support typically involves offering advice, practical tips, and coping techniques aimed at helping people to cope with cessation and the withdrawal of nicotine. It also explains how to use smoking cessation medications effectively [16]. It can be delivered through a range of modalities (face to face, digital, telephone, and group). It is widely implemented in practice, where it is delivered across care settings (primary care, stop smoking services, and secondary care), at varying levels of intensity, by a wide range of care providers (e.g., doctors, nurses, trained advisors, and pharmacists) [17] to a wide range of patient and public groups. It is a prime example of a complex intervention that is arguably susceptible to variable delivery in practice. Smoking is a priority target behaviour for behaviour change interventions and a public health priority due to its role in reinforcing health inequalities (Public Health [18]).

Behavioural support for smoking cessation has been implemented widely in clinical practice. In the UK, smoking cessation support is nationally available via the NHS stop smoking service, which offers free weekly support, nicotine replacement therapies, and other medicated aids [19]. Smoking cessation services have been shown to be highly effective [20]. However, outcomes across services are highly variable [21]. Recent studies looking at fidelity in NHS stop smoking services have shown that on average, approximately half of the intervention that is specified in the manual is delivered by stop smoking practitioners [22], representing low fidelity. This has also been found to be similar within telephone smoking cessation behavioural support [23]. Reasons underpinning this are unclear. There are national guidelines outlining how these interventions should be delivered, as well as intervention manuals for individual services [24]. There is even evidence to show that practitioners within the same service operating under the same treatment manual can have variable success rates [25], raising the possibility that the interventions are potentially delivered with variable degrees of fidelity.

All five stages of the BCC framework can be linked to smoking cessation interventions; the intervention may be designed and training offered at central NHS level but the delivery, receipt, and enactment take place within each smoking cessation service. Fidelity of design is important to ensure “that a study adequately tests its hypotheses in relation to its underlying theoretical and clinical processes” [26]. In the context of smoking cessation behavioural support, the logic model and/or treatment manual should contain intervention components or behaviour change techniques (BCTs) linked to the theory it claims to be based on.

Fidelity of training refers to “standardising training between providers, ensuring that providers are trained to criterion, and monitoring and maintaining provider skills over time” [26]. For smoking cessation interventions, this looks at whether stop smoking advisors were trained consistently, using standardised procedures. Was their training/acquired competence assessed prior to intervention delivery (i.e., by role playing delivering a session to a client/smoker and assessing whether delivered according to manual/as intended)?

Treatment delivery is defined as “treatment differentiation (did the providers only deliver the target treatment and not other treatments), treatment competency (did providers maintain the skill set learned in training), and treatment adherence (delivery of the treatment components as intended)” [26]. Within smoking cessation interventions, this looks at whether the intervention was delivered as intended/specified in manuals (e.g., audio-recording sessions).

Receipt refers to “whether or not the participant understood the treatment (as well as the accuracy of understanding) and demonstrates knowledge of, and ability to use, the skills or recommendations learned in treatment” [26]. This involves trying to ascertain whether the smoker understood during the session what they need to do prior to the quit attempt, e.g., prepare themselves by obtaining nicotine replacement therapy (NRT), removing cues to smoking from their house (such as ash trays), amongst others.

Enactment “involves assessment, monitoring, and improving the ability of participants to perform treatment-related behavioural skills and cognitive strategies in relevant real-life settings” [26]. In smoking cessation, this refers to whether smokers take the necessary steps to aid their quit attempt, e.g., use the NRT as directed. NIH was chosen as it is intended to synthesise other existing frameworks and is thus arguably comprehensive and represents a fidelity pathway to outcomes.

Reviews of other specific behaviours have been conducted, but there has been no review to date investigating fidelity assessments of smoking cessation interventions and the methods used in cases where it has been assessed. This is crucial given that research shows smoking cessation interventions are often delivered with variable degrees of fidelity [22].

### 1.2. Research Aims and Objectives

This review is aimed at investigating how fidelity has been assessed in studies that claim to investigate it. Specifically, the review looked at how the five dimensions of the NIH BCC fidelity framework (design, training, delivery, receipt, and enactment) have been assessed and reported in RCTs of smoking cessation behavioural support interventions. Fidelity is important in aiding interpretation of trial outcomes. There has been no review to date investigating fidelity assessment within smoking cessation interventions. This has been broken down further into the following:

1. Which dimensions of the framework have been reported and assessed?
2. What methodological approaches have been used to collect and analyse fidelity data for each dimension?
3. To what extent were associations between fidelity outcomes and intervention outcomes (e.g., smoking cessation) investigated?

## 2. Methods

The review was conducted in accordance with the PRISMA systematic review guidelines [27].

### 2.1. Inclusion and Exclusion Criteria

Studies were eligible for inclusion in the review if they met the following criteria.

#### 2.1.1. Population

Interventions targeting smokers of any age were eligible, including adolescent/student populations.

#### 2.1.2. Intervention

Only smoking cessation behavioural interventions were included. This included smoking in terms of tobacco cigarettes, shisha or water pipe smoking, and secondhand smoke. Interventions focusing solely on cannabis smoking were excluded. Studies with a behavioural support element with a human interaction component (i.e., face-to-face or telephone interactions) were included. Interventions solely featuring a distant modality of interaction (such as web/app-based materials) or pharmacological interventions (e.g., Champix) were excluded due to the absence of a human interaction component. Studies delivered with a provider and recipient interaction element (i.e., face to face, via telephone) have more scope for variability in fidelity across all five dimensions, particularly delivery, receipt, and enactment. In comparison, for digital interventions (where fidelity of delivery may be standardised), the variation lies in receipt and enactment. Intervention fidelity is still relevant to digital, but due to feasibility constraints, the review focused solely on those involving interaction between provider and recipient (face to face or telephone), as this is where there is the greatest scope for variation in fidelity and greater relevance of the NIH BCC dimensions.

#### 2.1.3. Study Design

The studies were required to report an assessment of intervention fidelity data, either mentioned in the abstract or assessed in the full text if it was unclear from the abstract. The BCC fidelity framework was designed for fidelity assessments of cluster/RCT designs. Therefore, only RCTs comparing the intervention against a control (i.e., no intervention, standard practice, and another intervention) were eligible. Studies that involved training staff to deliver smoking cessation interventions but reported no participant outcomes were also excluded as they did not detail the actual intervention delivery.

Studies published in English, in peer-reviewed journals, were included. Study protocols were included, as the primary interest of the review is methods. Research/conference abstracts were excluded. Only studies published post 2006 were included, following publication of the BCC framework in 2005.

### 2.2. Search Strategy

In December 2018, five databases were searched electronically: MEDLINE, EMBASE, Ovid Nursing Full Text Plus, CINAHL, and PsycINFO.

The search strategy included terms related to fidelity, smoking, cessation, and intervention (Table 1). Search terms were informed by previous systematic reviews of intervention fidelity [28] and Cochrane reviews on smoking cessation [29–31]. Terms for RCTs were adapted from Coppo et al. [32]. Terms within each category were combined using “OR” (i.e., smoking terms were combined as “smok^∗^” OR “tobacco”). These individual search strings were combined with “AND”.

The search strategy was validated by conducting an initial search and checking whether it retrieved a criterion paper identified during the scoping search (study 8).

### 2.3. Study Selection

Following deduplication, remaining entries were screened by the primary researcher (SB) at title and abstract level against the inclusion and exclusion criteria. The full text was screened for unclear studies and those fulfilling the inclusion criteria. For interrater reliability, the second researcher (FL) screened 10% of the studies at the abstract level and percentage agreement was calculated.

### 2.4. Data Extraction

A data extraction form was developed featuring five main sections: study characteristics, fidelity definitions, dimensions assessed, data collection, and analysis strategies. Study characteristics included the research question/aim, design, participant details, and the results/conclusion summary. Data was also extracted on intervention mode of delivery, intervention providers, and theoretical basis (use of theory/models of fidelity and/or behaviour change).

The assessment of fidelity incorporated the BCC checklist from Bellg et al. [1] and looked at each of the five dimensions (design, training, delivery, receipt, and enactment) in further detail (see Supplementary Table 3). For example, fidelity of enactment includes two subcomponents looking at the assessment of participant performing intervention skills and strategies used to do this. The studies were coded for both enhancement and assessment strategies.

The BCC guidance recommends using this as a checklist for scoring fidelity assessments (e.g., [33]). Each component should be rated as present, absent but should be present, or not applicable. Absent but should be present was defined as occurrences where the “treatment fidelity information was inappropriately omitted, preventing the coder from being able to accurately assess the scientific validity of the article” [6]. Categories were considered not applicable when “the particular treatment fidelity strategy was not applicable to the study in question,” e.g., studies that indicated abstinence would be assessed long term rather than during the intervention period (as indicated in the checklist) were marked as not applicable [6].

A percentage fidelity score was calculated by taking the number of fidelity strategies reported divided by the total number of applicable strategies for each dimension (e.g., a study with three of the four applicable delivery components present/reported would score 75% for delivery fidelity). Studies received 0 if the dimension was not assessed. Fidelity scores were classified as low (less than 50%), medium (51 to 79%), and high (80 to 100%) based on published criteria [26]. An overall fidelity score was also calculated (number of components present in all five dimensions divided by the total number of applicable components across all five dimensions).

Data extraction included the methods for collecting data fidelity (e.g., audio/video taping and provider checklists), the methods for analysing this data, and the methods for investigating the statistical association between fidelity and outcomes. For example, delivery studies may have reported the percentage of components delivered compared to the manual.

The data extraction form was piloted on the criterion paper (study 6) and amended as necessary. Full data extraction for all studies was completed by the primary researcher (SB). The interrater reliability was calculated for 4% of the total studies, and agreement was defined as both researchers agreeing on whether a particular aspect of the data extraction was present, e.g., whether the study provided information about treatment dose.

### 2.5. Data Synthesis

Where appropriate, quantitative data were summarised using descriptive statistics. Fidelity data analysis methods were summarised and described using narrative synthesis.

## 3. Results

### 3.1. Study Selection

**Figure 2:**
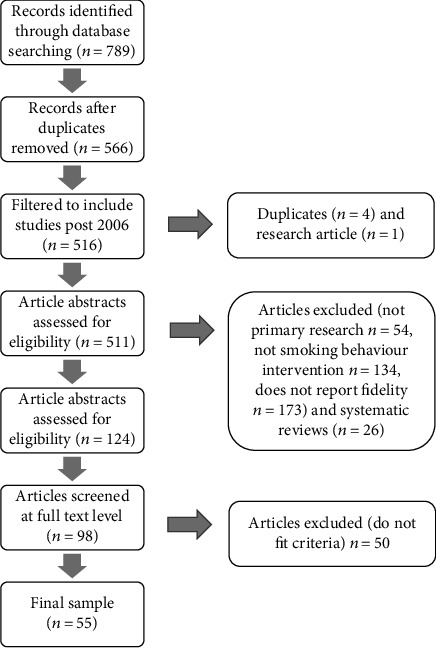
PRISMA diagram showing study selection.

The initial search yielded 789 studies, and 223 duplicates were removed (*n* = 566) (Figure 2). The studies were filtered to include studies post 2006 (*n* = 516). Five studies were removed (manual search for duplicates (*n* = 4) and one study was not original research (*n* = 1)). All 511 papers were screened at the abstract level. A second reviewer (FL) screened 7% of the 511 papers (*n* = 35), with 74% agreement between the two researchers. Any discrepancies were discussed until agreement was reached. 124 studies fit the eligibility criteria or were unclear and required further screening. Twenty-six systematic review papers were removed as they were not primary research.

The remaining 98 papers were screened at the full-text level, and 50 studies were excluded as they were not primary research. One study (study 11) described the findings from seven individual studies conducted within hospitals, which were treated individually. A final sample of 55 studies was included.

Interrater reliability was calculated on the data extraction for two studies (21, 28) with 82% agreement. Any discrepancies were discussed until agreement was reached.

### 3.2. Basic Study Characteristics

Table 2 outlines the summary characteristics (full details in Supplementary Table 1). The majority of the studies were conducted in the USA (*n* = 27, 49.1%). Most interventions were delivered within health settings (*n* = 27, 49.1%) and schools (*n* = 6, 10.9%). The most common intervention providers, accounting for more than half of the studies, were staff already delivering health interventions (e.g., diabetes educators) (*n* = 7, 12.7%), counsellors (*n* = 6, 10.9%), nurses (*n* = 5, 9.1%), and research staff/trained students (*n* = 4, 7.3%). Sample sizes varied, from 30 to 19,200 participants (average 1,554 participants). The most common mode and delivery was one-to-one/individual interventions (*n* = 36, 65.5%) and face-to-face delivery (*n* = 24, 63.6%).

### 3.3. Which Fidelity Dimensions Were Reported and Assessed (RQ1)?

The studies were assessed for fidelity in each of the five dimensions of the framework (design, training, delivery, receipt, and enactment). The percentages are the proportion of NIH BCC framework components listed for each of the five fidelity dimensions that are reported in individual papers.  Table 3 shows each fidelity dimension in rank order for average fidelity score across studies, how many components it contains, and the most and least reported component. The reporting of the fidelity subcomponents within each dimension was collated in full (Supplementary Table 2). The table shows enactment had the highest average fidelity score (92.7%) and training had the lowest (37.1%), indicating that enactment had the highest number of framework components present in studies conducting fidelity assessments.

Table 4 shows the overall level of observed fidelity across all five dimensions in each study, ranked from the lowest to highest scoring. The average fidelity score in terms of presence of framework components was 51.3% (range 14-83%), indicating that most studies reported observing low fidelity, as defined by Borrelli [26] (see Supplementary Table 2 for full results).

### 3.4. How Has Fidelity Been Assessed?

#### 3.4.1. Use of Theory and Frameworks

Almost half the studies (*n* = 24, 43%) did not cite the use of a theoretical framework (see Table 5 for summary). Motivational interviewing (MI) and Motivational Interviewing Treatment Integrity (MITI) scale were most commonly used in intervention design and to assess fidelity (MI *n* = 14, 25%; MITI *n* = 7, 12%). One study (5) used the RE AIM framework in addition to MITI, and another (18) used the fidelity protocol implementation index to assess fidelity. No studies cited the use of the BCC framework.

#### 3.4.2. Data Collection Methods

Fidelity data collection methods were not always reported, and where information was provided, methods were found to vary across and within fidelity dimensions. The most commonly used methods are shown in Table 6 (full data collection methods detailed in Supplementary Table 4). Many studies used multiple methods. Audiotaping and provider/participant self-reports were the most commonly used methods across all dimensions.

For the fidelity of design, only two studies specified using audiotaping, in addition to a checklist in the first study and in-person observation in the second. The remaining studies were unclear, and it was unclear how this data was analysed. For the fidelity of training, a variety of methods were used, including provider self-report (*n* = 3, 5%), audiotaping (*n* = 3, 5%), in-person observation (*n* = 7, 13%), role play (*n* = 7, 13%), meetings to discuss delivery (17), workshops (21), manuals (21), interviews (*n* = 3, 5%), study provider feedback (38), and consultations (43). For fidelity of delivery, studies collected fidelity data through audiotaping (*n* = 22, 40%) videotaping (study 6), checklists (*n* = 12, 28%), interviews (*n* = 6, 11%) in-person observation (*n* = 3, 5%), supervision (*n* = 7, 13%), online programme data (*n* = 2, 4%), protocol adherence data (17), and provider self-report data (*n* = 3, 5%). Collection of receipt data was often in terms of verifying skills and knowledge acquisition, with the majority of studies using participant self-reported questionnaires (*n* = 29, 53%), interviews (*n* = 7, 13%), or observations (*n* = 2, 4%). Fidelity of enactment data was often collected using self-reported questionnaires (*n* = 30, 55%) or provider checklists (*n* = 2, 4%).

#### 3.4.3. Groups Where Fidelity Was Assessed

The majority of studies assessed fidelity in the intervention group only (*n* = 37, 67%). The remaining studies assessed fidelity in both the intervention and control groups (*n* = 16, 29%). Almost half of the studies did not specify the proportion of their sample that fidelity was assessed in (*n* = 27, 49%). The remaining studies varied from 10% (*n* = 4, 7%) to 100% of the sample (*n* = 12, 22%) (average 55%). The study references are shown in Table 7, and full fidelity assessment data is shown in Supplementary Table 4.

#### 3.4.4. Measurement Time Points

The majority of studies measured fidelity at multiple time points (*n* = 20, 36%). 16 studies measured it at the end of the intervention (29%), eight during the intervention (15%), and one before and during the intervention (2%, study 5). It was not specified in the studies which fidelity dimensions were assessed at which time points. The majority of studies were unclear about the number of times fidelity was measured (*n* = 46, 84%). Four studies assessed fidelity on an ongoing basis (10%), eight once during the study (15%), and one assessed fidelity five times (2%, study 27). The study references are shown in Table 8, and full fidelity assessment data is shown in Supplementary Table 4.

#### 3.4.5. Fidelity Sampling Method

The majority of studies were unclear about their participant sampling method for the fidelity assessment (*n* = 32, 58%). The remaining studies either used purposive sampling (*n* = 2, 5%), random sampling (*n* = 8, 14%), or included the whole sample (*n* = 13, 24%). Two studies specified they were assessing fidelity amongst intervention providers in the sessions delivered, and two studies specified assessing fidelity in participant groups receiving the intervention. The study references are shown in Table 9, and full fidelity assessment data is shown in Supplementary Table 4.

### 3.5. How Was Fidelity Data Collected and Analysed (RQ2)?

Data was extracted on whether the studies assessed and reported findings of fidelity assessments for each dimension of the framework (Supplementary Table 5). Seven studies included were protocols, and so it was not possible to extract the reporting details (3, 5, 6, 8, 25, 30, and 43). These protocols may not have linked fidelity to outcomes because they did not have results as yet. For design, only one study (2%, 6) assessed design but did not report the findings. For training, eight studies (14.5%) assessed and reported the findings (5, 9, 21, 22, 24, 25, 37, and 38). For delivery, 35 studies (63.6%) assessed and reported the findings. For receipt, 31 studies (56.4%) assessed and reported the findings. Finally, for enactment, 27 studies (49.1%) assessed and reported the findings. The remaining studies in each dimension were unclear. This indicates that fidelity of delivery was the most commonly assessed and reported out of all five dimensions.

With regard to the approaches to fidelity analysis, the studies varied widely (Supplementary Tables 5 and 6). Methods for assessing and reporting the findings were described for each dimension as follows: design *n* = 1 study (1.8%), training *n* = 8 studies (14.5%), delivery *n* = 35 studies (63.6%), receipt *n* = 31 studies (56.4%), and enactment *n* = 27 studies (49.1%).

The majority of the studies did not analyse the fidelity data in any of the dimensions as they instead reported on strategies to enhance fidelity. For example, for training, two studies (16, 38) reported that the providers were continually trained and practised until they were able to deliver a session with fidelity. However, the assessment methods for this were not specified. In another study (37), the nurses (providers) were required to engage in role play to gain certification in the intervention procedures (fidelity of training). Finally, one study (43) provided feedback on recorded consultations. However, the nature of the feedback was unclear and it was unclear what they did with the role play data.

The delivery of the intervention was most commonly assessed for fidelity and the findings reported. The data was typically analysed by a staff member observing delivery of the intervention and completing a checklist of the components delivered or a self-reported checklist by intervention practitioners. The checklists were compared against intervention protocol, and scores were calculated to show delivery as outlined in the intervention protocol, e.g., using MITI checklists (studies 2, 5, 6, 9, 12, 14, 17, 18, 26–28, 30, 32, 34–39, 43–48, and 55). For receipt, participants were asked to complete questionnaires assessing usage and utility of intervention components in relation to their views on quitting smoking, for example (26). Questionnaires were also a commonly used method to assess enactment, e.g., using questionnaires to assess smoking reduction/quit behaviour and compare this to the fidelity of the intervention received (32). Design was rarely assessed or reported. Additional detail on the precise ratings/scoring of questionnaires was not reported for receipt or enactment data. It was unclear how the questionnaire data was used.

### 3.6. To What Extent Were Associations between Fidelity Outcomes and Intervention Outcomes Investigated (RQ3)?

All the studies did not report whether they had assessed reliability or validity. The majority of studies did not report whether they examined if there was an association between fidelity and study outcomes (*n* = 40, 73%). The remaining 15 studies (27%, studies 12, 19, 20, 23, 27, 32, 36, 39–41, 43, 46, 47, 49, and 55) used a variety of measures to assess this relationship. The majority of studies reported associations between participants' receptivity to intervention materials (fidelity of receipt) or usage of intervention components (fidelity of enactment) and smoking status/quit rates (studies 19, 20, 23, 27, 36, 40, 41, 43, and 49). Some studies explored the relationship between fidelity of delivery and a range of outcomes; one study looked at practitioners use of intervention materials in relation to quitting (43), another looked at practitioners' adherence to MI and smoking status (47), another assessed the predictive value of clients' characteristics on the practitioner's MI adherence (46), and another compared intervention conditions to study outcomes (55). The statistical analysis methodology used was not clearly stated in any of these studies, except in one study where odds ratios were calculated for reducing/quitting smoking and compared to the intervention delivery fidelity participants received from the practitioner (32). The studies reported greater positive outcomes with greater fidelity outcomes.

## 4. Discussion

This review is aimed at investigating how fidelity studies of smoking cessation behavioural support trials have assessed fidelity according to the five dimensions and recommendations of the NIH BCC fidelity framework (design, training, delivery, receipt, and enactment). The review looked at the methodology and analysis approaches used by studies to assess fidelity data and draw associations with the intervention outcomes.

55 studies were reviewed, and all the studies had low or medium overall fidelity in terms of the proportion of components recommended by the NIH BCC framework that were reported and assessed. The researchers discussed discrepancies on studies outlining training staff to deliver smoking cessation interventions but reported no participant outcomes and decided to exclude them as they were not RCTs. Enactment had the highest average fidelity score, and delivery was the most assessed and reported of the dimensions, with study staff completing checklists of the intervention components delivered and comparing it to the intervention protocol. This indicates that enactment had the highest number of components present but delivery was most commonly assessed and reported in studies conducting fidelity assessments. However, this could be due to the fact that outcome measurements for smoking cessation trials confound enactment, as the act of smoking cessation *is* enactment. As such, it would not necessarily be appropriate to explore the association between enactment and outcomes, as they are the same thing in this respect.

The main limitations of how fidelity is currently assessed for smoking cessation behavioural support trials are not investigating fidelity in both intervention and control arm. The studies stated that fidelity was assessed in the “intervention group only” or “intervention and control group,” so it was not possible to ascertain the exact nature of the fidelity assessment. This is a missed opportunity to explore contamination and treatment differentiation between trial arms. Another limitation is that none of the studies specified the sample used for conducting the fidelity assessment. The five dimensions of the framework each relate to a different part of the intervention; design, training, and delivery relate to the intervention providers, and receipt and enactment relate to the recipients. The vast majority of studies did not report whether there was an association between fidelity and study outcomes, indicating that when fidelity is measured it is not being interpreted with regard to the effect it has on study outcomes (i.e., helping participants to reduce/quit smoking). It is possible that the association was assessed but not reported.

### 4.1. Implications of Findings

The fidelity of delivery analysis may have been the most commonly assessed because of the existing emphasis on this dimension in the broader fidelity literature [34] and the often highlighted importance of this dimension in particular. It has been argued that assessing fidelity within intervention delivery is key to furthering understanding of the relationship between the intervention, the process, and the outcomes [34]. If an intervention is deemed to be ineffective at producing the results intended, the initial response may be to attribute that to a poorly designed intervention. However, the results may actually be due to a poor fit between the intervention design and delivery and the only way to deduce this is through assessing fidelity, known as a “type III error” [35], which refers to the concept of falsely dismissing a potentially effective intervention.

However, Borrelli et al. [6] would argue that it is important to assess fidelity from all aspects of the intervention process in order to highlight how loss of fidelity at the designer, provider, and/or recipient level can each impact on outcomes in different ways. This is required to assess whether an intervention is effective and if not, which aspects of it may be contributing to its ineffectiveness. Fidelity assessments in smoking cessation are largely unidimensional and often focus on investigating a single dimension of delivery, echoing findings from other systematic reviews of fidelity assessments (i.e., O'Shea et al. [13] and Lambert et al. [5]). When considering smoking behavioural support in particular, the healthcare professional is required to deliver a host of techniques so fidelity of delivery is arguably very important. However, whether or not these behaviour change techniques lead to behaviour change relies on various factors, namely, the client/smoker understanding these techniques and what they need to do. They must understand when their quit date will be (goal setting) and what they need to do to prepare for it (action planning) and then implement this (i.e., enactment, such as removing all cues to smoking such as ash trays and lighters (environmental restructuring) and obtaining their medication and starting it (pharmacological support)). If recipient level fidelity dimensions are not explored, then it is challenging to fully understand how and whether the intervention worked as intended.

The current review resonates with findings from other fidelity reviews in that all the studies had low or medium observed levels of fidelity [6, 12]. However, the present review differs in other respects. In Borrelli et al. [6] review, 27% of the studies reported checking study adherence against protocol. In this review, a higher percentage (62%) reported using a checklist to assess whether the intervention components were delivered as intended. This increase could be attributed to the nature of the studies. Borrelli et al. [6] looked at health behaviour change interventions overall (including multiple behaviours) whilst this review focused on a single behaviour of smoking cessation.

Other reviews focusing on specific aspects of the framework yielded similar results to this review. Rixon et al. [14] found fidelity of receipt was reported infrequently, whilst Walton et al. [15] looked at delivery and engagement with health behaviour change interventions and found it was most commonly measured. They also noted observational measures were the gold-standard methodology but that most studies used audiotaping and self-report questionnaires, as was the case in this review.

Many studies also reported fidelity enhancement strategies (such as regular supervision with practitioners) rather than fidelity assessment methods across all dimensions. The framework contains both fidelity enhancement and assessment items, and studies will vary in employing both. It is important to distinguish between the two strategies in order to understand whether the studies are assessing fidelity or assessing strategies that may lead to an increase in fidelity. Studies appear to be trying to enhance fidelity by considering fidelity at the study planning stages. However, this logically follows that fidelity should subsequently be assessed and reported to ascertain if the enhancement strategies increase fidelity but this review shows this is not routinely done.

None of the studies cited a fidelity theory or framework. This may in part help explain the limitations of current fidelity assessments for smoking cessation behavioural support, as they are not drawing on the best available guidance and conceptualisations of fidelity. In a study of 264 participants looking at barriers to researchers carrying out fidelity assessments, it was found that 89% indicated that fidelity is important. The majority of participants (68%) identified using strategies to assess fidelity (e.g., recording sessions) and enhancing fidelity (e.g., training manual), but only 30.9% indicated they reported these strategies within publications, noting the most common reason as being poor knowledge or understanding (77.4%). This indicates that researchers may have good awareness of the importance of intervention fidelity but poor knowledge and understanding is a barrier to addressing these in complex intervention trials [36].

### 4.2. Implications for Research

The key implications emerging from this review for consideration by intervention developers and researchers are that they should assess and report the following:

1. Details of fidelity in all stages of the intervention, from initial design through to participant enactment. This review showed most studies solely focused on delivery fidelity. Whilst this is important, the other aspects are equally important for assessing fidelity and linked outcomes
2. Fidelity assessment processes, such as where the fidelity sample has been drawn from. This review shows that fidelity assessment and measurement is not routinely reported and synthesising the evidence is difficult. Others planning or conducting fidelity assessments for similar studies cannot learn from existing evidence and methods and replicate. Inclusion of this would provide a clearer picture of fidelity in different stages of the overall process. Fidelity is included as an item in reporting guidelines (i.e., TIDiER, [37])
3. How fidelity measures and outcomes are linked. Only a small proportion of studies reported using checklists to score delivery, comparing this against the intervention protocol to check delivery was as intended and statistically assessed fidelity measures with outcome measures. The purpose of assessing fidelity is to aid the interpretation of the outcomes, and omitting this is a missed opportunity. It is also important to compare the intervention and control arms to look at treatment differentiation and contamination. This is particularly important in pragmatic trials evaluating interventions against usual/standard care which may have an active control arm (i.e., some behaviour change techniques) in order to maximise internal validity of trial and interpretation of outcomes. The majority of studies measured fidelity at multiple time points but did not specify which fidelity dimensions were assessed at which time points. This would have been an opportunity to explore fidelity drift/loss of sustainability within studies. Following similarly clear methodology from delivery across all dimensions and reporting the results demonstrate the correlation between fidelity levels and intervention outcomes
4. The difference between fidelity assessment and enhancement strategies. This review shows many studies are using the latter and thus trying to maximise fidelity during the trial. Whilst this is vital and should be planned for, it is important to also follow through and explore whether or not fidelity is maintained. A separate measure of fidelity is necessary and should logically follow to link fidelity to intervention outcomes

### 4.3. Strengths and Limitations

This study has a number of strengths and limitations. The use of the BCC framework has allowed for a comprehensive review of the literature using a framework that has unified previous fidelity models.

However, one limitation is the use of only published articles. Smoking cessation interventions are designed and delivered in a wide variety of settings and may well be evaluated and assessed for fidelity. They may also be assessed for cost-effectiveness to ascertain feasibility in a local context. These may be published as evaluation or programme reports, which are excluded from reviews of this nature.

Furthermore, it is possible that the studies used strategies to enhance fidelity and/or assessed fidelity but did not report it. This study is aimed at looking at what authors report in fidelity assessments. The studies reviewed varied greatly in their description of the interventions. This could be overcome by contacting study authors for further information or to understand whether they explored fidelity but did not report. However, this was beyond the scope of feasibility in the present study.

One potential limitation of this study is the use of the BCC framework to guide data extraction and analysis. There are multiple frameworks of fidelity that differ in how fidelity is defined and/or guidance for measurement (e.g., [10, 11]), but the current review highlights the utility of the BCC framework for assessing fidelity. NIH was chosen as it is intended to synthesise other existing frameworks and is thus arguably comprehensive and represents a fidelity pathway to outcomes. Interrater reliability was also assessed in a very small percentage of studies (4%), decreasing possible reliability.

Furthermore, the five dimensions each have varying numbers of subcomponents within them. It is easier to score higher if there are fewer components, as the overall percentage increases quicker. Future research could look at weighting the components to allow for a more equal comparison.

### 4.4. Future Research

Future research could benefit from focusing and addressing the implications above to ensure more accurate reporting of fidelity and subsequently a more accurate interpretation of the effectiveness of an intervention. Further research is needed to identify whether the dimensions within the framework differ in their importance and effect on intervention outcomes. For example, perhaps delivery fidelity was most commonly reported due to the ease of assessing and reporting this dimension compared to others. This could potentially be used to offer guidance on how to measure and report fidelity for interventions where a comprehensive fidelity assessment using the framework may not be feasible, such as in local authority public health settings.

### 4.5. Conclusions

The review looked at which dimensions of the framework have been reported and assessed. It highlighted that fidelity evaluations in smoking cessation behavioural support interventions are not comprehensively exploring fidelity at the intervention designer, provider, and recipient levels. Providers focus predominantly on assessing fidelity of delivery and enactment. There is wide variability in methodological and analytical approaches that precludes comparison and synthesis across studies. Many studies reported assessing numerous components of fidelity; however, the findings/results were not reported in turn. This represents a waste of research effort and lack of transparency. Findings have highlighted recommendations for improving fidelity evaluations and reporting practices, such as ensuring studies are using fidelity assessments to aid interpretation of the outcomes.

## Figures and Tables

**Table 1:** Search terms used to conduct electronic searches.

Fidelity terms		Smoking terms		Cessation terms		Intervention terms		RCT
Fidelity	AND	Smok^∗^	AND	Cessation	AND	Intervention^∗^	AND	Randomised control trial
OR		OR		OR		OR		OR
Intervention adheren^∗^		*Tobacco*		Quit^∗^		Treatment		Randomized control trial
OR				OR		OR		OR
Integrity				Control		Counsel^∗^		Controlled clinical trial
OR				OR		OR		OR
Intervention implement^∗^				Stop		Program^∗^		*Clinical trial*
OR						OR		OR
Intervention complian^∗^						Strategy^∗^		Meta analysis
OR						OR		
Process evaluation						Support		
OR						OR		
Intervention deliver^∗^						Behaviour change^∗^		
OR								
Engag^∗^								

Italicised terms are MESH terms.

**Table 2:** Study characteristics.

	Number of studies	Percentage of total studies (*n* = 55)
*Country*		
US	27	49.1
UK	5	9.1
Germany	4	7.3
Netherlands	3	5.5
India	1	1.8
Australia	1	1.8
Spain	1	1.8
*Setting*		
Health system	27	49.1
School	6	10.9
Community	4	7.3
Telephone	2	3.6
College	1	1.8
University	1	1.8
Leaflets	1	1.8
*Intervention providers*		
Staff delivering health interventions (e.g., diabetes educators and smoking cessation advisors)	7	12.7
Counsellors	6	10.9
Nurses	5	9.1
Research staff/trained students	4	7.3
Hospital staff (doctors, nurses, assistants, etc.)	3	5.5
Computer/web based	2	3.6
Teachers	2	3.6
Doctors	1	1.8
Psychologist	1	1.8
Health professionals	1	1.8
Teachers and student peer leaders	1	1.8
Therapist	1	1.8
Peer counsellors	1	1.8
Other school staff (drug education officers)	1	1.8
Pharmacist	1	1.8
Health trainers	1	1.8
Leaflets	1	1.8
Unclear	3	5.5
*Intervention recipients*		
School pupils	7	12.7
Patients in hospital	6	10.9
Smokers (including those not motivated or ready to quit)	4	7.3
Pregnant smokers	3	5.5
Patients at GP surgeries	2	3.6
Teenagers in hospital	2	3.6
Nurses who smoke and primary caregivers of children	1	1.8
Patients with familial hypocholesteraemia	1	1.8
Patients with diabetes	1	1.8
Smokers with attention deficit hyperactivity disorder (ADHD)	1	1.8
Nurses and inpatient smokers	1	1.8
Ethnic minority pregnant smokers	1	1.8
Smokers and nonsmoking pairs living with a child	1	1.8
Ethnic minority smokers	1	1.8
Heavy smokers	1	1.8
Overweight smokers	1	1.8
Nurses	1	1.8
Cancer survivors	1	1.8
Adults planning to stay quit post discharge	1	1.8
Adults	1	1.8
Women recently given birth	1	1.8
Smokers wanting to quit	1	1.8
Undergraduate students	1	1.8
Smokers with low motivation to quit	1	1.8
*Format*		
One to one	36	65.5
Group	5	9.1
Unclear	1	1.8
*Mode of delivery*		
Face to face	24	43.6
Telephone	7	12.7
Face to face and digital	4	7.3
Face to face and media	3	5.5
Face to face and telephone	2	3.6
Face to face, digital, and telephone	1	1.8
Unclear	1	1.8

**Table 3:** Reporting of fidelity components within each dimension, in rank order.

Fidelity dimension	Average fidelity score (%) (range)	Total number of components in NIH BCC fidelity framework	Most reported component (*n* studies, % of total review sample)	Least reported component (*n* studies, % of total review sample)
Enactment	92.7% (0–100%)	2	Participant performance of the intervention skills will be assessed in settings in which the intervention might be applied (*n* = 53, 96%)	A strategy will be used to assess performance of the intervention skills in settings in which the intervention might be applied) (*n* = 51, 93%)

Design	56.1% (5–95%)	7	Information about the treatment dose in the intervention condition (*n* = 51, 93%)	Plans to address possible setbacks in implementation (i.e., backup systems or providers) (*n* = 13, 24%)

Receipt	48% (0–100%)	5	The participants' ability to perform the intervention skills being assessed during the intervention period (*n* = 50, 91%)	Multicultural factors considered in the development and delivery of the intervention (e.g., provided in native language; protocol is consistent with the values of the target group) (*n* = 7, 13%)

Delivery	44.5% (0–77%)	9	The method to ensure that the content of the intervention is delivered as specified (*n* = 47, 85%)	Whether there was a plan for the assessment of whether or not proscribed components were delivered (e.g., components that are unnecessary or unhelpful) (*n* = 1, 2%)

Training	37.1% (0–100%)	7	Description of how providers will be trained (*n* = 42, 76%)	Presence of a training plan that takes into account trainees' different education and experience and learning styles (*n* = 1, 2%)

Average fidelity score refers to the presence of framework components.

**Table 4:** Table showing overall fidelity in studies, ranked from low to high fidelity.

	Study (author/year)	Overall (% of *n* components present out of all possible applicable components; 43 max)
6	Buhse 2013	86.00%
3	Bock 2014	83.70%
20	Gilbert 2017	79.07%
22	Gould 2018	79.07%
1	Asfar 2018	72.09%
2	Blaakman 2013	69.80%
8	Catley 2012	69.80%
21	Goenka 2010	69.80%
26	Horn 2008	69.80%
17	Duffy 2015 USCD	67.40%
30	Lycett 2010	67.40%
44	Taskila 2012	67.40%
45	Taylor 2014	67.40%
47	Thyrian Freyer 2010	67.40%
10	Dahne 2018	65.12%
43	Spanou 2010	62.80%
28	Kealey 2009	60.50%
34	Mujika 2014	60.50%
35	Park 2006	60.50%
54	White 2017	60.47%
46	Thyrian Freyer 2007	58.10%
31	Matthews 2018	55.81%
36	Parker 2007	55.80%
37	Pbert Fletcher 2006	55.80%
48	Toll 2010	55.80%
14	Duffy 2015 NYU	51.20%
32	McCambridge 2008	51.20%
38	Pbert, Osganian 2006	51.20%
42	Sloboda 2009	51.20%
33	McClure 2017	51.16%
5	Broekhuizen 2010	48.80%
12	Duffy 2015 KU	48.80%
50	Varvel 2010	48.80%
55	Windsor 2014	48.80%
9	Croghan 2012	46.50%
25	Harter 2015	44.20%
53	Webb 2007	44.20%
16	Duffy 2015 UMMC	41.90%
18	El-Mohandes 2013	41.90%
40	Schlam 2018	37.21%
49	Unrod 2016	37.21%
51	Wang 2017	37.21%
52	Wang 2018	37.21%
11	Duffy 2015 KPCHR	34.90%
39	Richter 2016	34.90%
41	Schulz 2014	34.90%
4	Bonevski 2016	34.88%
19	Escoffery 2016	32.60%
23	Haas 2015	30.20%
29	Leung 2017	27.91%
27	Johnson 2009	27.90%
13	Duffy 2015 MGH	23.30%
7	Busch 2015	20.90%
24	Halcomb 2015	20.90%
15	Duffy 2015 UAB	14.00%
	Average % (range)	51.33% (14-83)

**Table 5:** Theoretical and methodological frameworks.

Theoretical framework or theory	Number of studies (*n* = 55 max)	Study reference numbers
MI	14	5, 8, 24, 26, 28, 32, 34–36, 45–47, 50
MITI	7	2, 5, 28, 32, 34, 46, 47
Based on interventions shown to be effective in Cochrane review	7	11–17
Social cognitive theory	8	1, 3, 19, 21, 28, 33, 38, 45
Cognitive behavioural therapy	3	3, 28, 33
I change model of behaviour change	2	5, 41
Stages of change theory	4	19, 38, 46, 49
RE AIM framework	1	5
Fidelity protocol implementation index (PII)	1	18
Process assessment framework	1	21
Chronic care model	1	23
Social contextual model for reducing tobacco use	1	23
Behaviour change taxonomy	3	20, 22, 30
Control theory	1	45
Self-determination theory	1	45
5A model recommended by the US Public Health Service clinical practice guideline and the American Academy of Pediatrics	2	10, 37
System changes' approach	1	4, 19
Ziedonis' ATTOC model	1	4
Theoretical domain framework	1	22
AWARD (ask, warn, advise, refer, and do it again)	2	51, 52

**Table 6:** Data collection methods.

BCC framework dimension	Most commonly used reporting methods (*n*, % of total studies)
Design	Audiotaping and observation (*n* = 1, 1.8%) (study 28)
	Audiotaping and checklist (*n* = 1, 1.8%) (study 2)

Training	In-person observation (*n* = 7, 13%) (studies 6, 8, 12, 16, 17, 26, and 28)
	Role play (*n* = 7, 13%) (studies 9, 14, 21, 38, and 42–44)
	Self-report (*n* = 3, 5%) (studies 2, 12, and 36)
	Interviews (*n* = 3, 5%) (studies 24, 25, and 30)
	Audiotaping (*n* = 3, 5%) (studies 5, 43, and 48)

Delivery	Audiotaping (*n* = 22, 40%) (studies 2, 3, 5, 8, 9, 13, 14, 20, 28, 30, 32–36, 39, and 43–48)
	Checklists (*n* = 12, 28%) (studies 3, 11, 12, 14, 16, 18, 21, 26, 27, 34, 37, and 54)
	Interviews (*n* = 6, 11%) (studies 7, 24, 25, 30, 44, and 45)
	In-person observation (*n* = 3, 5%) (studies 8, 12, and 42)
	Supervision (*n* = 7, 13%) (studies 8, 9, 11, 35, and 46–48)
	Online programme data (*n* = 2, 4%) (studies 13 and 15)
	Protocol adherence data (*n* = 1, 2%) (study 17)
	Provider self-report data (*n* = 3, 5%) (studies 38, 42, and 50)

Receipt	Participant self-reported questionnaire (*n* = 29, 53%) (studies 1, 3, 4, 5, 10, 14, 16, 17, 20, 22, 26, 27, 29–32, 35, 37, 38, 40, 42, 43, 46, 48–50, and 53–55)
	Interviews (*n* = 7, 13%) (studies 1, 3, 19, 25, 37, 45, and 54)
	Observations (*n* = 2, 4%) (studies 2 and 9)

Enactment	Participants self-reported questionnaire (*n* = 30, 55%) (studies 1, 3–6, 16, 17, 20, 22, 25–27, 29, 30–33, 35, 37, 38, 40–43, 46, 48–50, 53, and 54)
	Interviews (*n* = 5, 9%) (studies 1, 3, 19, 37, and 45)
	Provider checklists (*n* = 2, 4%) (studies 12 and 21)

**Table 7:** Fidelity assessment.

Group where fidelity was assessed (*n*, % of total studies)	Study references
Fidelity assessed in the intervention group only (*n* = 37, 65%)	Studies 2, 3, 5–9, 11–15, 18, 19, 21, 23–28, 30, 34–37, 39, 41–44, 46–48, 50, 54, and 55
Fidelity assessed in both the intervention and control groups (*n* = 16, 29%)	Studies 12, 16, 17, 32, 38, 45, and 53
Studies that specified the proportion of the sample that fidelity was assessed in (*n* = 19, 35%) This varied from 10% (*n* = 4, 7%) to 100% of the sample (*n* = 12, 22%) (average 55%).	Studies 2, 3, 5, 12, 16, 19, 23, 24, 34–37, 39, 42, 44, 45, 47, 50, and 53

**Table 8:** Fidelity measurement time points.

Fidelity measurement time points (*n*, % of total studies)	Study references
End of the intervention (*n* = 16, 29%)	Studies 1, 7, 12, 18, 19, 20, 23–26, 31, 40, 49–52
During the intervention (*n* = 8, 15%)	Studies 9, 11, 34, 36, 38, 44, 48, and 54
Fidelity assessed on an ongoing basis (*n* = 4, 10%)	Studies 3, 35, 42, and 45
Fidelity assessed once during the study (*n* = 8, 15%)	Studies 1, 23–25, 28, 49, 51, and 52

**Table 9:** Fidelity sampling method.

Fidelity sampling method (*n*, % of total studies)	Study references
Purposive sampling (*n* = 2, 5%)	Studies 44 and 46
Random sampling (*n* = 8, 14%)	Studies 2, 3, 9, 14, 20, 28, 33, and 34
Included the whole sample (*n* = 13, 24%)	Studies 23, 24, 35, 37, 40, 42, 45, and 49–54
Specified they were assessing fidelity amongst intervention providers in the sessions delivered (*n* = 2, 5%)	Studies 42 and 47
Specified assessing fidelity in participant groups receiving the intervention (*n* = 2, 5%)	Studies 43 and 44

## Data Availability

Data is available as supplementary data tables.

## Appendix

### List of studies included in the review with the study IDs

1. Asfar, T., Caban-Martinez, A., McClure, L., Ruano-Herreria, E., Sierra, D., Gilford Clark, G., Samano, D., Dietz, N., Ward, K., Arheart, K. and Lee, D. (2018). A cluster randomized pilot trial of a tailored worksite smoking cessation intervention targeting Hispanic/Latino construction workers: Intervention development and research design. Contemporary Clinical Trials, 67, pp.47-55.

2. Blaakman S., Tremblay P.J., Halterman J.S., Fagnano M., & Borrelli, B. (2013). Implementation of a community-based secondhand smoke reduction intervention for caregivers of urban children with asthma: Process evaluation, successes and challenges. Health Education Research, 28(1), 141-152.

3. Bock B.C., Rosen R.K., Fava J.L., Gaskins R.B., Jennings E., Thind H., . . . Marcus, B. H. (2014). Testing the efficacy of yoga as a complementary therapy for smoking cessation: Design and methods of the BreathEasy trial. Contemporary Clinical Trials, 38(2), 321-332.

4. Bonevski, B., Guillaumier, A., Shakeshaft, A., Farrell, M., Tzelepis, F., Walsberger, S., . . . Skelton, E. (2016). An organisational change intervention for increasing the delivery of smoking cessation support in addiction treatment centres: study protocol for a randomized controlled trial. Trials, 17, 1.

5. Broekhuizen K., van Poppel M.N., Koppes L.L., Brug J., & van Mechelen, W. (2010). A tailored lifestyle intervention to reduce the cardiovascular disease risk of individuals with familial hypercholesterolemia (FH): Design of the PRO-FIT randomised controlled trial. BMC Public Health, 10, 69.

6. Buhse S., Heller T., Kasper J., Muhlhauser I., Muller U.A., Lehmann T., & Lenz, M. (2013). An evidence-based shared decision making programme on the prevention of myocardial infarction in type 2 diabetes: Protocol of a randomised-controlled trial. BMC Family Practice, 14, 155.

7. Busch V., De Leeuw J.R., Zuithoff N.P., Van Yperen T.A., & Schrijvers, A. J. (2015). A controlled health promoting school study in the Netherlands: Effects after 1 and 2 years of intervention. Health Promotion Practice, 16(4), 592-600.

8. Catley D., Harris K.J., Goggin K., Richter K., Williams K., Patten C., . . . Liston, R. (2012). Motivational interviewing for encouraging quit attempts among unmotivated smokers: Study protocol of a randomized, controlled, efficacy trial. BMC Public Health, 12, 456.

9. Croghan I.T., Trautman J.A., Winhusen T., Ebbert J.O., Kropp F.B., Schroeder D.R., & Hurt, R. D. (2012). Tobacco dependence counseling in a randomized multisite clinical trial. Contemporary Clinical Trials, 33(4), 576-582.

10. Dahne, J., Wahlquist, A. E., Boatright, A. S., Garrett-Mayer, E., Fleming, D. O., Davis, R., . . . Carpenter, M. J. (2018). Nicotine replacement therapy sampling via primary care: Methods from a pragmatic cluster randomized clinical trial. Contemporary Clinical Trials, 72.

11. Duffy S.A., Cummins S.E., Fellows J.L., Harrington K.F., Kirby C., Rogers E., . . . Rigotti, N. (2015). Fidelity monitoring across the seven studies in the consortium of hospitals advancing research on tobacco (CHART). Study at Kaiser Permanente Center for Health Research (KPCHR). Tobacco Induced Diseases, 13(1) (pagination), Arte Number: 29. ate of Pubaton: 03 Se 2015.

12. Duffy S.A., Cummins S.E., Fellows J.L., Harrington K.F., Kirby C., Rogers E., . . . Rigotti, N. (2015). Fidelity monitoring across the seven studies in the consortium of hospitals advancing research on tobacco (CHART). Study at University of Kansas (KU). Tobacco Induced Diseases, 13(1) (pagination), Arte Number: 29. ate of Pubaton: 03 Se 2015.

13. Duffy S.A., Cummins S.E., Fellows J.L., Harrington K.F., Kirby C., Rogers E., . . . Rigotti, N. (2015). Fidelity monitoring across the seven studies in the consortium of hospitals advancing research on tobacco (CHART). Study at Massachusetts General Hospital (MGH). Tobacco Induced Diseases, 13(1) (pagination), Arte Number: 29. ate of Pubaton: 03 Se 2015.

14. Duffy S.A., Cummins S.E., Fellows J.L., Harrington K.F., Kirby C., Rogers E., . . . Rigotti, N. (2015). Fidelity monitoring across the seven studies in the consortium of hospitals advancing research on tobacco (CHART). Study at New York University (NYU). Tobacco Induced Diseases, 13(1) (pagination), Arte Number: 29. ate of Pubaton: 03 Se 2015.

15. Duffy S.A., Cummins S.E., Fellows J.L., Harrington K.F., Kirby C., Rogers E., . . . Rigotti, N. (2015). Fidelity monitoring across the seven studies in the consortium of hospitals advancing research on tobacco (CHART). Study at University of Alabama at Birmingham (UAB). Tobacco Induced Diseases, 13(1) (pagination), Arte Number: 29. ate of Pubaton: 03 Se 2015.

16. Duffy S.A., Cummins S.E., Fellows J.L., Harrington K.F., Kirby C., Rogers E., . . . Rigotti, N. (2015). Fidelity monitoring across the seven studies in the consortium of hospitals advancing research on tobacco (CHART). Study at University of Michigan Medical Center (UMMC). Tobacco Induced Diseases, 13(1) (pagination), Arte Number: 29. ate of Pubaton: 03 Se 2015.

17. Duffy S.A., Cummins S.E., Fellows J.L., Harrington K.F., Kirby C., Rogers E., . . . Rigotti, N. (2015). Fidelity monitoring across the seven studies in the consortium of hospitals advancing research on tobacco (CHART). Study at University of California, San Diego (UCSD). Tobacco Induced Diseases, 13(1) (pagination), Arte Number: 29. ate of Pubaton: 03 Se 2015.

18. ElMohandes A.A., Windsor R., Tan S., Perry D.C., Gantz M.G., & Kiely, M. (2013). A randomized clinical trial of trans-dermal nicotine replacement in pregnant African American smokers. Maternal and Child Health Journal, 17(5), 897-906.

19. Escoffery, C., Bundy, L., Haardoerfer, R., Berg, C. J., Savas, L. S., Williams, R. S., & Kegler, M. C. (2016). A process evaluation of an intervention to promote home smoking bans among low income households. Evaluation and Program Planning, 55, 120-125. 10.1016/j.evalprogplan.2015.12.008

20. Gilbert, H., Sutton, S., Morris, R., Petersen, I., Wu, Q., Parrott, S., . . . Nazareth, I. (2017). Start2quit: a randomised clinical controlled trial to evaluate the effectiveness and cost-effectiveness of using personal tailored risk information and taster sessions to increase the uptake of the NHS Stop Smoking Services. Health technology assessment, 21, 3.

21. Goenka S., Tewari A., Arora M., Stigler M.H., Perry C.L., Arnold J.P., . . . Reddy, K. S. (2010). Process evaluation of a tobacco prevention program in Indian schools--methods, results and lessons learnt. Health Education Research, 25(6), 917-935.

22. Gould, G. S., Bar-Zeev, Y., Bovill, M., Atkins, L., Gruppetta, M., Clarke, M. J., & Bonevski, B. (2017). Designing an implementation intervention with the Behaviour Change Wheel for health provider smoking cessation care for Australian Indigenous pregnant women. Implementation Science, 12, 1.

23. Haas, J. S., Linder, J. A., Park, E. R., Gonzalez, I., Rigotti, N. A., Klinger, E. V., . . . Williams, D. R. (2015). Proactive tobacco cessation outreach to smokers of low socioeconomic status: A randomized clinical trial. JAMA Internal Medicine, 175(2), 218-226. 10.1001/jamainternmed.2014.6674

24. Halcomb, E. J., Furler, J. S., Hermiz, O. S., Blackberry, I. D., Smith, J. P., Richmond, R. L., & Zwar, N. A. (2015). Process evaluation of a practice nurse-led smoking cessation trial in Australian general practice: Views of general practitioners and practice nurses. Family Practice, 32(4), 468-473. 10.1093/fampra/cmv041

25. Harter M., Bartsch A.L., Egger N., Konig H.H., Kriston L., Schulz H., . . . Buchholz, A. (2015). Evaluating a collaborative smoking cessation intervention in primary care (ENTER): Study protocol for a cluster-randomized controlled trial. Trials, 16(1) (pagination), Arte Number: 447. ate of Pubaton: October 09, 2015.

26. Horn K., Dino G., Hamilton C., Noerachmanto N., & Zhang, J. (2008). Feasibility of a smoking cessation intervention for teens in the emergency department: Reach, implementation fidelity, and acceptability. American Journal of Critical Care: An Official Publication, American Association of Critical-Care Nurses, 17(3), 205-216.

27. Johnson C.C., Myers L., Webber L.S., Boris N.W., He H., & Brewer, D. (2009). A school-based environmental intervention to reduce smoking among high school students: The Acadiana coalition of teens against tobacco (ACTT). International Journal of Environmental Research and Public Health, 6(4), 1298-1316.

28. Kealey K.A., Ludman E.J., Marek P.M., Mann S.L., Bricker J.B., & Peterson, A. V. (2009). Design and implementation of an effective telephone counseling intervention for adolescent smoking cessation. Journal of the National Cancer Institute, 101(20), 1393-1405.

29. Leung, W., Roberts, V., Gordon, L. G., Bullen, C., McRobbie, H., Prapavessis, H., . . . Maddison, R. (2017). Economic evaluation of an exercise-counselling intervention to enhance smoking cessation outcomes: The Fit2Quit trial. Tobacco Induced Diseases, 15.

30. Lycett D., Hajek P., & Aveyard, P. (2010). Trial protocol: Randomised controlled trial of the effects of very low calorie diet, modest dietary restriction, and sequential behavioural programme on hunger, urges to smoke, abstinence and weight gain in overweight smokers stopping smoking. Trials, 11(pagination), Arte Number: 94. ate of Pubaton: 07 Ot 2010

31. Matthews, A. K., Steffen, A., Kuhns, L., Ruiz, R., Ross, N., Burke, L., . . . King, A. C. (2018). Evaluation of a randomized clinical trial comparing the effectiveness of a culturally targeted and non-targeted smoking cessation intervention for lesbian, gay, bisexual and transgender (LGBT) smokers. Nicotine and Tobacco Research.

32. McCambridge J., Slym R.L., & Strang, J. (2008). Randomized controlled trial of motivational interviewing compared with drug information and advice for early intervention among young cannabis users. Addiction, 103(11), 1809-1818.

33. McClure, J. B., Blasi, P. R., Cook, A., Bush, T., Fishman, P., Nelson, J., . . . Catz, S. L. (2017). Oral health 4 life: Design and methods of a semi-pragmatic randomized trial to promote oral health care and smoking abstinence among tobacco quitline callers. Contemporary Clinical Trials.

34. Mujika, A., Forbes, A., Canga, N., de Irala, J., Serrano, I., Gascó, P., & Edwards, M. (2014). Motivational interviewing as a smoking cessation strategy with nurses: An exploratory randomised controlled trial. International Journal of Nursing Studies, 51(8), 1074-1082. 10.1016/j.ijnurstu.2013.12.001

35. Park, E. R., Puleo, E., Butterfield, R. M., Zorn, M., Mertens, A. C., Gritz, E. R., . . . Emmons, K. M. (2006). A process evaluation of a telephone-based peer-delivered smoking cessation intervention for adult survivors of childhood cancer: The partnership for health study. Preventive Medicine, 42(6), 435-442.

36. Parker, D. R., Windsor, R. A., Roberts, M. B., Hecht, J., Hardy, N. V., Strolla, L. O., & Lasater, T. M. (2007). Feasibility, cost, and cost-effectiveness of a telephone-based motivational intervention for underserved pregnant smokers. Nicotine & Tobacco Research: Official Journal of the Society for Research on Nicotine and Tobacco, 9(10), 1043-1051.

37. Pbert L., Osganian S.K., Gorak D., Druker S., Reed G., O'Neill K.M., & Sheetz, A. (2006). A school nurse-delivered adolescent smoking cessation intervention: A randomized controlled trial. Preventive Medicine, 43(4), 312-320.

38. Pbert, L., Fletcher, K. E., Flint, A. J., Young, M. H., Druker, S., & DiFranza, J. (2006). Smoking prevention and cessation intervention delivery by pediatric providers, as assessed with patient exit interviews. Pediatrics, 118(3), e810-24.

39. Richter, K. P., Faseru, B., Shireman, T. I., Mussulman, L. M., Nazir, N., Bush, T., . . . Martell, M. J. (2016). Warm handoff versus fax referral for linking hospitalized smokers to quitlines. American Journal of Preventive Medicine, 51(4), 587-596. 10.1016/j.amepre.2016.04.006

40. Schlam, T. R., Cook, J. W., Baker, T. B., Hayes-Birchler, T., Bolt, D. M., Smith, S. S., . . . Piper, M. E. (2018). Can we increase smokers adherence to nicotine replacement therapy and does this help them quit? Psychopharmacology, 235, 7.

41. Schulz D.N., Kremers S.P., Vandelanotte C., van Adrichem M.J., Schneider F., Candel M.J., & de Vries, H. (2014). Effects of a web-based tailored multiple-lifestyle intervention for adults: A two-year randomized controlled trial comparing sequential and simultaneous delivery modes. Journal of Medical Internet Research, 16(1), e26.

42. Sloboda, Z., Stephens, R. C., Stephens, P. C., Grey, S. F., Teasdale, B., Hawthorne, R. D., . . . Marquette, J. F. (2009). The adolescent substance abuse prevention study: A randomized field trial of a universal substance abuse prevention program. Drug & Alcohol Dependence, 102(1-3), 1-10. 10.1016/j.drugalcdep.2009.01.015

43. Spanou, C., Simpson, S. A., Hood, K., Edwards, A., Cohen, D., Rollnick, S., . . . Butler, C. C. (2010). Preventing disease through opportunistic, rapid engagement by primary care teams using behaviour change counselling (PRE-EMPT): Protocol for a general practice-based cluster randomised trial. BMC Family Practice, 11, 10p-10p. 10.1186/1471-2296-11-69

44. Taskila T., Macaskill S., Coleman T., Etter J.F., Patel M., Clarke S., . . . Aveyard, P. (2012). A randomised trial of nicotine assisted reduction to stop in pharmacies - the RedPharm study. BMC Public Health, 12, 182.

45. Taylor, A. H., Thompson, T. P., Greaves, C. J., Taylor, R. S., Green, C., Warren, F. C., . . . West, R. (2014). A pilot randomised trial to assess the methods and procedures for evaluating the clinical effectiveness and cost-effectiveness of exercise assisted reduction then stop (EARS) among disadvantaged smokers. Health Technology Assessment (Winchester, England), 18(4), 1-324. 10.3310/hta18040

46. Thyrian J.R., FreyerAdam J., Hannover W., Roske K., Mentzel F., Kufeld C., . . . Hapke, U. (2007). Adherence to the principles of motivational interviewing, clients' characteristics and behavior outcome in a smoking cessation and relapse prevention trial in women postpartum. Addictive Behaviors, 32(10), 2297-2303.

47. Thyrian J.R., FreyerAdam J., Hannover W., Roske K., Mentzel F., Kufeld C., . . . Hapke, U. (2010). Population-based smoking cessation in women post partum: Adherence to motivational interviewing in relation to client characteristics and behavioural outcomes. Midwifery, 26(2), 202-210.

48. Toll B.A., Martino S., Latimer A., Salovey P., O'Malley S., CarlinMenter S., . . . Cummings, K. M. (2010). Randomized trial: Quitline specialist training in gain-framed vs standard-care messages for smoking cessation. Journal of the National Cancer Institute, 102(2), 96-106.

49. Unrod, M., Simmons, V. N., Sutton, S. K., Cummings, K. M., Celestino, P., Craig, B. M., . . . Brandon, T. H. (2016). Relapse-prevention booklets as an adjunct to a tobacco quitline: A randomized controlled effectiveness trial. Nicotine and Tobacco Research, 18, 3.

50. Varvel S.J., Cronk N.J., Harris K.J., & Scott, A. B. (2010). Adaptation of a lay health advisor model as a recruitment and retention strategy in a clinical trial of college student smokers. Health Promotion Practice, 11(5), 751-759.

51. Wang, M. P., Li, W. H., Cheung, Y. T., Lam, O. B., Wu, Y., Kwong, A. C., . . . Lam, T. H. (2018). Brief advice on smoking reduction versus abrupt quitting for smoking cessation in Chinese smokers: A cluster randomized controlled trial. Nicotine and Tobacco Research, 20, 1.

52. Wang, M. P., Suen, Y. N., Li, W. H.-C., Lam, C. O.-B., Wu, S. Y.-d., Kwong, A. C.-S., . . . Lam, T. H. (2017). Intervention With Brief Cessation Advice Plus Active Referral for Proactively Recruited Community Smokers: A Pragmatic Cluster Randomized Clinical Trial. JAMA Internal Medicine, 177, 12.

53. Webb, M. S., Hendricks, P. S., & Brandon, T. H. (2007). Expectancy priming of smoking cessation messages enhances the placebo effect of tailored interventions. Health Psychology, 26(5), 598-609.

54. White, J., Hawkins, J., Madden, K., Grant, A., Er, V., Angel, L., ... & Midgley, L. (2017). Adapting the ASSIST model of informal peer-led intervention delivery to the Talk to FRANK drug prevention programme in UK secondary schools (ASSIST?+ FRANK): intervention development, refinement and a pilot cluster randomised controlled trial. Public Health Research, 5(7), 1-126.

55. Windsor, R., Clark, J., Cleary, S., Davis, A., Thorn, S., Abroms, L., & Wedeles, J. (2014). Effectiveness of the smoking cessation and reduction in pregnancy treatment (SCRIPT) dissemination project: A science to prenatal care practice partnership. Maternal & Child Health Journal, 18(1), 180-190. 10.1007/s10995-013-1252-7

## Supplementary Materials

Supplementary Materials Supplementary Table 1: study characteristics. Supplementary Table 2: reporting of BCC framework dimensions. Supplementary Table 3: reporting of individual BCC framework dimensions. Supplementary Table 4: fidelity measurement methodology. Supplementary Table 5: approaches to fidelity analysis and associations with intervention outcomes. Supplementary Table 6: analysis of fidelity data.
