# Supplementary material for: How Has Intervention Fidelity Been Assessed in Smoking Cessation Interventions? A Systematic Review
Source: J Smok Cessat. 2021 Jan 15;2021:6641208. doi: 10.1155/2021/6641208 (PMC8279202; doi:10.1155/2021/6641208)
Supplement: Supplementary Materials — Supplementary Table 1: study characteristics. Supplementary Table 2: reporting of BCC framework dimensions. Supplementary Table 3: reporting of individual BCC framework dimensions. Supplementary Table 4: fidelity measurement methodology. Supplementary Table 5: approaches to fidelity analysis and associations with intervention outcomes. Supplementary Table 6: analysis of fidelity data. [file 6641208.f1.docx]

# Supplementary Table 1 Study characteristics

|  | **Study (author/year)** | **Country** | **Setting** | **Intervention Providers** | **Recipients** | **Total sample size (N)** | **Format** | **Mode of delivery** |
| --- | --- | --- | --- | --- | --- | --- | --- | --- |
|  | Asfar 2018 | United States | Work place | Not clear | Male,≥18 years old, Hispanic/Latino, and have smoked ≥5 cigarettes/day for the past year  They need access to a telephone, no plans to move in the next six months, be interested in making a serious quit attempt in the next 30 days, and have no contraindication to NRT. | Total: 126 (across 14 sites)  Intervention group: 63 Control group: 63 | Individual (one to one) | Face to face |
|  | Blaakman 2013 | US | Other (School) | Nurses | - Nurses who smoked at large teaching hospital in Spain - Primary care givers of smoke exposed children aged 3-10 | Total: 530 Intervention group: 140 Control group: not reported | Individual (one to one) | Telephone |
|  | Bock 2014 | US | Community | Psychologist with experience in conducting smoking cessation groups | Smokers | Total: 300 Intervention group: 150 Control group: 150 | Group | Face to face |
|  | Bonevski 2016 | Australia | Health system | Existing governmental and non-governmental drug and alcohol treatment centres | Participants aged over 16 years who are attending their first of a number of visits (to allow for repeated exposure to the intervention), who are self-reported current smokers, sufficiently proficient in English, and do not have severe untreated mental illness | Total: 33 centres  Intervention group: unclear Control group: unclear | Individual (one to one) | Face to Face and Telephone |
|  | Broekhuizen 2010 | Netherlands | Community | Computer delivered digital intervention plus Motivational Interviewing delivered in recipient homes by lifestyle coach | Participants are individuals who were diagnosed with Familial Hypercholesterolemia. | Total sample Size: 400 Intervention group: 200 Control group: 200 | Individual (one to one) | Face to face  Digital |
|  | Buhse 2013 | Germany | Health System (Diabetes Clinic) | Diabetes educators | Patients with diabetes | Total sample Size: 154 Intervention group: 77 Control group: 77 | Group | Face to face |
|  | Busch 2015 | Netherlands | School | Teachers | School Pupils | Total sample Size of study: 969  Intervention group 2 schools, approx. 700 students each Control group 2 schools, approx. 700 students each | Unclear | Unclear |
|  | Catley 2012 | US | Community | Master’s level health professionals with prior training and experience using MI | Current smokers in the general population who are not currently motivated or ready to quit | Total sample Size of study: 255 Intervention group not known | Individual (one to one) | Face to face |
|  | Croghan 2012 | US | Health System | Interventionists trained to use Smoke Free  and Living It manual | Smokers with attention deficit hyperactivity disorder (ADHD) | Total 255 | Individual (one to one) | Face to face |
|  | Dahne 2018 | United States | Health system | Bachelors level study staff with Treatment Specialist (TTS) training | 1) age 18+, 2) smoker of at least five cigarettes per day on ≥25 days out of the last 30 days, 3) English speaking, and 4) recruited through a primary care site actively enrolled in the study.  Exclusion criteria included FDA contraindications for NRT use | Total 22 clinics  Intervention group: 10 clinics Control group: 12 clinics | Individual (one to one) | Face to face |
|  | Duffy 2015 KPCHR | US | Health System | Unclear | Patients in hospital | Total 900 Intervention group, 599 Control group 301 | Individual (one to one) | Face to face Web |
|  | Duffy 2015 KU | US | Health System | Counsellors | Patients in hospital | Total 1054 | Individual (one to one) | Face to face |
|  | Duffy 2015 MGH | US | Health System | Unclear | Patients in hospital | Total 529 | Individual (one to one) | Face to face Web |
|  | Duffy 2015 NYU | US | Health System | Counsellors | Patients in hospital | Total 1619 Intervention group 805 Control group 814 | Individual (one to one) | Face to face |
|  | Duffy 2015 UAB | US | Health System | Counselor | Patients | Total 1488 Intervention group 748 Control group 740 | Individual (one to one) | Face to face Web Telephone |
|  | Duffy 2015 UMMC | US | Health System | Hospital nurses | Nurses and inpatient smokers | Intervention group 1528 | Individual (one to one) | Face to face |
|  | Duffy 2015 USCD | US | Health System | Quitline staff | Inpatient smokers | Total 1270 | Individual (one to one) | Face to face |
|  | El-Mohandes 2013 | US | Health System | Unclear | 30 weeks pregnant women self-identified as an ethnic minority, smoker with a desire to quit. | Total sample Size: 52 Intervention group: 26 Control group: 26 | Individual (one to one) | Face to face |
|  | Escoffery 2016 | US | Other (Telephone) | Research staff | Smokers and non smokers (a combination of at least one smoker and one non-smoker in the home (including children)) | Total sample Size: 498 Intervention group: 249 Control group: 249 | Individual (one to one) | Telephone |
|  | Gilbert 2017 | UK | Health system | Smoking cessation advisors in stop smoking services | Current smokers aged ≥ 16 years, able to read English, motivated to quit and who had not attended the SSS in the previous 12 months | Total: 4384 Intervention group: 2636 Control group: 1748 | Individual (one to one) | Face to face/Letters |
|  | Goenka 2010 | India | Other (School) | Teachers and student peer leaders | Pupils | Total sample size 16 schools with 5564 students | Group | Other (Poster) |
|  | Gould 2018 | Australia | Health system | Healthcare professionals working with women | Pregnant women who were current smokers, were up to 28-weeks' gestation, aged 16 years or over, and expecting an Indigenous baby | Total: unclear Intervention group: 22 smokers Control group: unclear | Individual (one to one) | Face to face |
|  | Haas 2015 | US | Health System | Tobacco treatment specialists | Smokers who described their race and/or ethnicity as black, Hispanic, or white | Total sample Size of study: 8544 Intervention group 4436 Control group 4108 | Individual (one to one) | Telephone |
|  | Halcomb 2015 | Australia | Health System | Practice nurse | Patients at GP surgeries | Total sample Size of study: 101 practices with 2390 patients Intervention group 876 Control group 678, quitline 836 | Individual (one to one) | Face to face |
|  | Harter 2015 | Germany | Health System | Doctors | Heavy smokers (smoke at least 20 cigarettes per day) and/or suffer from COPD or cardiovascular disease. | Total sample Size of study: 800 patients across 40 surgeries Intervention group 800 | Individual (one to one) | Face to face |
|  | Horn 2008 | US (West Virginia) | Health System | Trained adult professionals | Teen participants were patients 14 to 19 years old who came to the emergency department for care for any reason during a 2-year period (2002-2004). Teens were eligible for the intervention if they reported smoking on 1 or more days in the preceding 30 days | Total sample Size of study: 76 Intervention group 40 Control group 34 | Individual (one to one) | Face to face |
|  | Johnson 2009 | US | Other (High School) | Teachers | 10 schools with 9th grade students | Total sample Size of study 20 schools Intervention group 10 schools, 4763 students Control group 10 schools, students not reported | Group | Face to face School media campaign and teachers also spoke to them |
|  | Kealey 2009 | US | Health System | Counselors | School pupils | Intervention 25 experimental schools, 948 pupils Control group 330 | Individual (one to one) | Telephone |
|  | Leung 2017 | New Zealand | Community | Trained exercise-facilitators (participant- support person [PSPs]) | Unclear | Total: 906 Intervention group: 455 Control group: 451 | Individual (one to one) | Telephone |
|  | Lycett 2010 | UK | Health System | Trained practice nurses. | Overweight (BMI > 25 Kg/m2) smokers | Total sample Size of study: 90 (30 per group in three arms) Intervention group 30 Control group 30 | Individual (one to one) | Face to face |
|  | Matthews 2018 | United States | Community | LGBT- and non- LGBT–identified research staff | Participants who (1) self-identity as LGBT, (2) age 18–65, (3) current smoker (more than five packs in lifetime AND past year smoking AND 4 or more days per week AND carbon monoxide [CO] expired-air reading of ≥8 ppm), (4) greater than or equal to 5 on a 10-point Likert scale measuring desire to quit smoking, and (6) no prior adverse reactions to nicotine replacement patches | Total: 345 Intervention group: 172 Control group: 173 | Group | Face to Face |
|  | McCambridge 2008 | UK | College | Researcher practitioners | Students aged 16–19 who used cannabis at least weekly | Total sample Size: 270 Intervention group: 135 Control group: 135 | Individual (one to one) | Face to face |
|  | McClure 2017 | United States | Community | Alere Wellbeing (AW) research counsellors, AW is standard quit line provider | Callers were deemed potentially eligible if they smoked at least 5 cigarettes a day, were age 18 or older, were ready to quit smoking in the next month, could read and speak in English, and were eligible for their state's multi-call treatment program | Total: 718 Intervention group: 358 Control group: 360 | Individual (one to one) | Telephone and Leaflet |
|  | Mujika 2014 | Spain | Health system | Therapist | Smoking nurses who worked at a university hospital | Total 30, Intervention group 15 Control group 15 | Individual (one to one) | Face to face |
|  | Park 2006 | US | Health System (Cancer Institute) | Peer counselors (childhood cancer survivors) | Childhood cancer survivors who were current smokers | Total sample Size: 796 Intervention group: 398 Control group: 398 | Individual (one to one) | Telephone |
|  | Parker 2007 | US | Health System | Trained counsellors | Female smoker no more than 26 weeks pregnant | Total sample Size of study: 1065  Intervention group two groups, 329 and 358. Results here for 358  Control group 378 | Individual (one to one) | Face to face Telephone |
|  | Pbert Fletcher 2006 | US | Health System (Paediatric Unit) | Hospital staff (48 pediatricians, 10 nurse practitioners, 1 physician assistant, and 2 pediatric residents) | Adolescents patients 13 to 17 years of age regardless of smoking status | Total sample Size of study: 2710 | Individual (one to one) | Face to face |
|  | Pbert, Osganian 2006 | US | Other (School) | School nurses | All adolescents in participating high schools who reported using tobacco on at least 1 day in the past 30 days and were interested in quitting in the next 2 weeks | Total sample Size: 1148 Intervention group 571 Control group 577 | Individual (one to one) | Face to face |
|  | Richter 2016 | US | Health System | Quitline staff | Adult patients planning to stay quit post-discharge | Total sample Size of study: 1054 Intervention group 527 Control group 527 | Individual (one to one) | Telephone |
|  | Schlam 2018 | United States | Health system | Case managers (bachelor’s level study staff supervised by a licensed clinical psychologist) delivered the treatment in patients’ primary care clinics | Adult smokers attending any clinic outpatient visit who were smoking ≥ 5 cigarettes per day over the past 6 months; not using varenicline or bupropion; and having no medical contraindications to nicotine replacement therapy (NRT) | Total: 513 | Individual (one to one) | Face to face |
|  | Schulz 2014 | Netherlands | Community | Web-based computer-tailored program | Adults with internet access | Total sample Size of study: 4833 Intervention group 1828, 1765 Control group 1797 | Individual (one to one) | Web |
|  | Sloboda 2009 | US | Other (School) | School Drug Abuse Resistance Education (D.A.R.E.) officers. | Pupils | Total sample Size of study: 19,200 pupils Intervention: 11,118 Control: 8082 | Group | Face to face |
|  | Spanou 2010 | UK | Health System | One GP and one practice nurse per surgery | GP patients | Total sample Size of study: 1104 with 24 practices recruiting 46 patients | Individual (one to one) | Face to face |
|  | Taskila 2012 | UK | Health System | Pharmacists who work for NHS stop smoking services | Adult daily smokers who smoke at least 10 cigarettes or 8 g of loose tobacco as “roll up” cigarettes daily and don’t intend to stop in the next month, but are prepared to reduce their consumption with any of the programmes offered. | Total sample Size of study: 160 (ESTIMATED SAMPLE SIZE, THIS IS PROTOCOL) | Individual (one to one) | Face to face |
|  | Taylor 2014 | UK | Health System | Health Trainers | Adult smokers smoking at least 10 cigarettes per day (and had done so for at least 2 years), did not want to quit in the next month and did not wish to use NRT to reduce smoking | Total sample Size of study: 99 Intervention group 49 Control group 50 | Individual (one to one) | Face to face Telephone |
|  | Thyrian Freyer 2007 | Germany | Health System (Maternity Ward of Hospitals) | Counselors (trained experts in MI) | Women who indicated that they had smoked prior to pregnancy | Total sample Size of study: 869 Intervention group 418 Control group 451 | Individual (one to one) | Face to face |
|  | Thyrian Freyer 2010 | Germany | Health System | Graduate students trained in MI | Women who had recently given birth at maternity ward who smoked. | Total sample Size of study: 84 | Individual (one to one) | Face to face |
|  | Toll 2010 | US | Telephone Smoking Cessation | Smokers’ Quitline staff | Smoker seeking support to quit | Total sample Size of study: 2032 Intervention group 810 Control group 1222 | Individual (one to one) | Telephone |
|  | Unrod 2016 | United States | Community | New York State Smokers’ Quitline (NYSSQL) | Clients of the New York State Smokers’ Quitline (NYSSQL) who (1) smoked at least 10 cigarettes per day over the month prior to calling the quitline; (2) at least 18 years old; (3) able to speak and read English; and (4) reached by the NYSSQL at their 2-week call back | Total: 3458 Intervention group: Massed mailing (n = 1127), Repeated mailing (n = 1142) Control group: 1189 | Individual (one to one) | Telephone/Leaflets |
|  | Varvel 2010 | US | Other (University setting) | Lay health advisors LHAs (students and members of community) | Undergraduate students | Total sample Size of study: 118 Intervention group 118 | Individual (one to one) | Face to face |
|  | Wang 2017 | Hong Kong | Community | University students (health-related studies) and volunteers from nongovernmental organizations | Adults (aged ≥18 years) who had smoked at least 1 cigarette a day in the past 3months, exhaled carbon monoxide of 4ppm or more, expressed the intention to quit or reduce smoking, had a local telephone number for follow-up, were not participating in other SC programs, and were physically and mentally able to communicate in Cantonese | Total: 1226 Intervention group: active referral (n = 402), brief advice (n = 416)  Control (n = 408) | Individual (one to one) | Face to face |
|  | Wang 2018 | Hong Kong | Community | Trained smoking cessation ambassadors | Smokers who joined the Quit and Win Contest in 2014, adult smokers from the community in all 18 districts of Hong Kong. Smokers who had smoked at least 1 cigarette daily in the past 3 months, had an expiratory carbon monoxide (CO) concentration of at least 4 ppm and expressed a willingness to reduce or quit smoking | Total: 1077 Intervention group: cut down to quit (CDTQ) (n = 559), quit immediately (n = 518) | Individual (one to one) | Face to face |
|  | Webb 2007 | US | Other (Leaflets) | NA, intervention involved providing leaflets | Smokers with low to moderate motivation to quit | Total sample Size of study: N  standard/no prime (n 77), the standard/ prime (n 70), the placebo tailoring/no prime (n 73), or the placebo tailoring/prime (n 69) | Individual (one to one) | Other (Leaflets) |
|  | White 2017 | South Wales | School | Year 8 School students, peer training | Other school students | Total: 1567  Intervention group: +FRANK 419, FRANK Friends 440, Assist 347  Control group: 361 | Group | Face to face / web |
|  | Windsor 2014 | US | Health System | Designated Care Coordinators (DCC) who are nurses and social workers | Pregnant women | Total sample Size of study: 518 Intervention group 259 Control group 259 | Individual (one to one) | Face to face |

# Supplementary Table 2 Reporting of BCC framework dimensions

|  | **Study (author/year)** | **Fidelity of Design**  **(% of n components present out of all possible applicable components; 20 max)** | **Fidelity of Training (% of n components present out of all possible applicable components; 7 max)** | **Fidelity of Delivery (% of n components present out of all possible applicable components; 9 max)** | **Fidelity of Receipt (% of n components present out of all possible applicable components; 5 max)** | **Fidelity of Enactment (% of n components present out of all possible applicable components; 2 max)** | **OVERALL (% of n components present out of all possible applicable components; 43 max)** |
| --- | --- | --- | --- | --- | --- | --- | --- |
| 1 | Asfar 2018 | 90.00% | 0.00% | 66.67% | 100.00% | 100.00% | 72.09% |
| 2 | Blaakman 2013 | 80.00% | 57.10% | 55.60% | 60.00% | 100.00% | 69.80% |
| 3 | Bock 2014 | 95.00% | 71.40% | 77.80% | 60.00% | 100.00% | 83.70% |
| 4 | Bonevski 2016 | 60.00% | 14.29% | 0.00% | 20.00% | 50.00% | 34.88% |
| 5 | Broekhuizen 2010 | 45.00% | 42.90% | 33.30% | 80.00% | 100.00% | 48.80% |
| 6 | Buhse 2013 | 85.00% | 100.00% | 77.80% | 80.00% | 100.00% | 86.00% |
| 7 | Busch 2015 | 10.00% | 14.30% | 22.20% | 40.00% | 100.00% | 20.90% |
| 8 | Catley 2012 | 85.00% | 71.40% | 55.60% | 20.00% | 100.00% | 69.80% |
| 9 | Croghan 2012 | 30.00% | 57.10% | 55.60% | 60.00% | 100.00% | 46.50% |
| 10 | Dahne 2018 | 80.00% | 42.86% | 55.56% | 40.00% | 100.00% | 65.12% |
| 11 | Duffy 2015 KPCHR | 25.00% | 28.60% | 44.40% | 40.00% | 100.00% | 34.90% |
| 12 | Duffy 2015 KU | 50.00% | 28.60% | 55.60% | 40.00% | 100.00% | 48.80% |
| 13 | Duffy 2015 MGH | 15.00% | 14.30% | 33.30% | 20.00% | 100.00% | 23.30% |
| 14 | Duffy 2015 NYU | 55.00% | 57.10% | 55.60% | 0.00% | 100.00% | 51.20% |
| 15 | Duffy 2015 UAB | 20.00% | 14.30% | 11.10% | 0.00% | 0.00% | 14.00% |
| 16 | Duffy 2015 UMMC | 30.00% | 42.90% | 55.60% | 40.00% | 100.00% | 41.90% |
| 17 | Duffy 2015 USCD | 80.00% | 42.90% | 66.70% | 40.00% | 100.00% | 67.40% |
| 18 | El-Mohandes 2013 | 50.00% | 14.30% | 33.30% | 40.00% | 100.00% | 41.90% |
| 19 | Escoffery 2016 | 35.00% | 0.00% | 33.30% | 40.00% | 100.00% | 32.60% |
| 20 | Gilbert 2017 | 95.00% | 71.43% | 66.67% | 40.00% | 100.00% | 79.07% |
| 21 | Goenka 2010 | 85.00% | 57.10% | 55.60% | 40.00% | 100.00% | 69.80% |
| 22 | Gould 2018 | 90.00% | 71.43% | 66.67% | 60.00% | 100.00% | 79.07% |
| 23 | Haas 2015 | 40.00% | 0.00% | 0.00% | 60.00% | 100.00% | 30.20% |
| 24 | Halcomb 2015 | 30.00% | 28.60% | 11.10% | 0.00% | 0.00% | 20.90% |
| 25 | Harter 2015 | 35.00% | 28.60% | 55.60% | 60.00% | 100.00% | 44.20% |
| 26 | Horn 2008 | 90.00% | 28.60% | 44.40% | 80.00% | 100.00% | 69.80% |
| 27 | Johnson 2009 | 5.00% | 28.60% | 44.40% | 60.00% | 100.00% | 27.90% |
| 28 | Kealey 2009 | 55.00% | 71.40% | 55.60% | 60.00% | 100.00% | 60.50% |
| 29 | Leung 2017 | 50.00% | 0.00% | 0.00% | 20.00% | 50.00% | 27.91% |
| 30 | Lycett 2010 | 75.00% | 57.10% | 44.40% | 80.00% | 100.00% | 67.40% |
| 31 | Matthews 2018 | 60.00% | 42.86% | 44.44% | 60.00% | 100.00% | 55.81% |
| 32 | McCambridge 2008 | 60.00% | 14.30% | 44.40% | 60.00% | 100.00% | 51.20% |
| 33 | McClure 2017 | 70.00% | 0.00% | 44.44% | 40.00% | 100.00% | 51.16% |
| 34 | Mujika 2014 | 55.00% | 57.10% | 55.60% | 80.00% | 100.00% | 60.50% |
| 35 | Park 2006 | 55.00% | 57.10% | 55.60% | 80.00% | 100.00% | 60.50% |
| 36 | Parker 2007 | 55.00% | 42.90% | 55.60% | 60.00% | 100.00% | 55.80% |
| 37 | Pbert Fletcher 2006 | 50.00% | 71.40% | 33.30% | 80.00% | 100.00% | 55.80% |
| 38 | Pbert, Osganian 2006 | 50.00% | 28.60% | 55.60% | 60.00% | 100.00% | 51.20% |
| 39 | Richter 2016 | 40.00% | 0.00% | 33.30% | 40.00% | 100.00% | 34.90% |
| 40 | Schlam 2018 | 60.00% | 0.00% | 0.00% | 40.00% | 100.00% | 37.21% |
| 41 | Schulz 2014 | 40.00% | 0.00% | 33.30% | 40.00% | 100.00% | 34.90% |
| 42 | Sloboda 2009 | 50.00% | 71.40% | 55.60% | 0.00% | 100.00% | 51.20% |
| 43 | Spanou 2010 | 70.00% | 57.10% | 55.60% | 40.00% | 100.00% | 62.80% |
| 44 | Taskila 2012 | 65.00% | 71.40% | 66.70% | 60.00% | 100.00% | 67.40% |
| 45 | Taylor 2014 | 65.00% | 71.40% | 66.70% | 60.00% | 100.00% | 67.40% |
| 46 | Thyrian Freyer 2007 | 75.00% | 14.30% | 44.40% | 60.00% | 100.00% | 58.10% |
| 47 | Thyrian Freyer 2010 | 65.00% | 71.40% | 66.70% | 60.00% | 100.00% | 67.40% |
| 48 | Toll 2010 | 55.00% | 57.10% | 55.60% | 40.00% | 100.00% | 55.80% |
| 49 | Unrod 2016 | 55.00% | 0.00% | 22.22% | 40.00% | 50.00% | 37.21% |
| 50 | Varvel 2010 | 40.00% | 42.90% | 55.60% | 60.00% | 100.00% | 48.80% |
| 51 | Wang 2017 | 60.00% | 14.29% | 0.00% | 40.00% | 50.00% | 37.21% |
| 52 | Wang 2018 | 60.00% | 0.00% | 0.00% | 40.00% | 100.00% | 37.21% |
| 53 | Webb 2007 | 60.00% | 0.00% | 33.30% | 40.00% | 100.00% | 44.20% |
| 54 | White 2017 | 65.00% | 42.86% | 66.67% | 40.00% | 100.00% | 60.47% |
| 55 | Windsor 2014 | 35.00% | 57.10% | 66.70% | 40.00% | 100.00% | 48.80% |
|  | AVERAGE % (RANGE) | 56.09% (5-95) | 37.14% (0-100) | 44.45% (0-77) | 48.00% (0-100) | 92.73% (0-100) | 51.33% (14-83) |

## Supplementary Table 3 Reporting of individual BCC framework dimensions

| BCC Framework components | Studies assessed for which component was applicable (n, total n = 55) | Studies assessed for which component was applicable (%) |
| --- | --- | --- |
| **Treatment Design** |  |  |
| 1. Provide information about treatment dose in the intervention condition | 51 | 93% |
| a) Length of contact (minutes) | 36 | 65% |
| b) Number of contacts | 49 | 89% |
| c) Content of treatment | 52 | 95% |
| d) Duration of contact over time | 48 | 87% |
| 2. Provide information about treatment dose in the comparison condition | 33 | 60% |
| a) Length of contact (minutes) | 20 | 36% |
| b) Number of contacts | 26 | 47% |
| c) Content of treatment | 30 | 55% |
| d) Duration of contact over time | 28 | 51% |
| e) Method to ensure that dose is equivalent between conditions. | 14 | 25% |
| f) Method to ensure that dose is equivalent for participants within conditions | 10 | 18% |
| 3. Specification of provider credentials that are needed. | 37 | 67% |
| 4. Theoretical model upon which the intervention is based is clearly articulated | 31 | 56% |
| a) The active ingredients are specified and incorporated into the intervention | 31 | 56% |
| b) Use of experts or protocol review group to determine whether the intervention protocol reflects the underlying theoretical model or clinical guidelines | 16 | 29% |
| c) Plan to ensure that the measures reflect the hypothesized theoretical constructs/mechanisms of action | 22 | 40% |
| 5. Potential confounders that limit the ability to make conclusions at the end of the trial are identified. | 47 | 85% |
| 6. Plan to address possible setbacks in implementation (i.e., backup systems or providers) | 13 | 24% |
| 7. If more than one intervention is described, all described equally well | 28 | 51% |
| **Training Providers** |  |  |
| 1. Description of how providers will be trained (manual of training procedures) | 42 | 76% |
| 2. Standardization of provider training (especially if multiple waves of training are needed for multiple groups of providers). | 20 | 36% |
| 3. Assessment of provider skill acquisition. | 28 | 51% |
| 4. Assessment and monitoring of provider skill maintenance over time | 22 | 40% |
| 5. Characteristics being sought in a treatment provider are articulated a priori. Characteristics that should be avoided in a treatment provider are articulated a priori. | 22 | 40% |
| 6. At the hiring stage, assessment of whether or not there is a good fit between the provider and the intervention (e.g., ensure that providers find the intervention acceptable, credible, and potentially efficacious | 8 | 15% |
| 7. There is a training plan that takes into account trainees’ different education and experience and learning styles. | 1 | 2% |
| **Delivery of Treatment** |  |  |
| 1. Method to ensure that the content of the intervention is delivered as specified. | 47 | 85% |
| 2. Method to ensure that the dose of the intervention is delivered as specified. | 42 | 76% |
| 3. Mechanism to assess if the provider actually adhered to the intervention plan or in the case of computer delivered interventions, method to assess participants’ contact with the information. | 44 | 80% |
| 4. Assessment of nonspecific treatment effects. | 2 | 4% |
| 5. Use of treatment manual. | 29 | 53% |
| 6. There is a plan for the assessment of whether or not the active ingredients were delivered. | 41 | 75% |
| 7. There is a plan for the assessment of whether or not proscribed components were delivered. (e.g., components that are unnecessary or unhelpful) | 1 | 2% |
| 8. There is a plan for how will contamination between conditions be prevented | 12 | 22% |
| 9. There is an a priori specification of treatment fidelity (e.g., providers adhere to delivering >80% of components). | 6 | 11% |
| **Receipt of Treatment** |  |  |
| 1. There is an assessment of the degree to which participants understood the intervention. | 47 | 85% |
| 2. There are specification of strategies that will be used to improve participant comprehension of the intervention. This is about tactics they may have used to ensure participants acquire knowledge and skills, such as practice during the intervention session, reminders etc | 15 | 27% |
| 3. The participants’ ability to perform the intervention skills will be assessed during the intervention period. | 50 | 91% |
| 4. A strategy will be used to improve subject performance of intervention skills during the intervention period. | 15 | 27% |
| 5. Multicultural factors considered in the development and delivery of the intervention (e.g., provided in native language; protocol is consistent with the values of the target group). | 7 | 13% |
| **Enactment of Treatment Skills** |  |  |
| 1. Participant performance of the intervention skills will be assessed in settings in which the intervention might be applied. | 53 | 96% |
| 2. A strategy will be used to assess performance of the intervention skills in settings in which the intervention might be applied. | 51 | 93% |

# Supplementary table 4 Fidelity measurement methodology

|  | **Study author/year** | **Theoretical framework cited (fidelity or health psychology theory)** | **Data collection method(s)** | | | | | **Fidelity assessment sample** | **What proportion of sample was fidelity assessed in?** | **When in relation to intervention?** | **How many times was fidelity measured?** | **How was fidelity sample selected?** |
| --- | --- | --- | --- | --- | --- | --- | --- | --- | --- | --- | --- | --- |
|  |  |  | **Design** | **Training** | **Delivery** | **Receipt** | **Enactment** |  |  |  |  |  |
| 1 | Asfar 2018 | Social cognitive model facilitators, our formative data, along with the extant treatment literature for cessation | Unclear | Unclear | Participant self-reported questionnaire / Interviews with P | Participant self-reported questionnaire / Interviews with P | Participant self-reported questionnaire / Interviews with P | Intervention and control group | Intervention: unclear  Control: 25 p, approx 40% (interviews)  Survey unclear | At the end of the study only | Once | Unclear |
| 2 | Blaakman 2013 | Motivational Interviewing Treatment Integrity for delivery | Audiotaping, Checklist | Provider self-report (checklist) | Audiotaping | In person observation, Telephone Calls | In person observation, Telephone Calls | Intervention group only (recipients and providers) | 20% | Unclear | Unclear | Randomly |
| 3 | Bock 2014 | Yes Health Psychology CBT and SCT for the design and delivery of the smoking cessation intervention | Unclear | Unclear | Audiotaping, Nurses completed  intervention checklists for 100% of MI sessions | Participant self-reported questionnaire Other -interviews with p | Participant self-reported questionnaire Other -Interviews with p | Intervention group only (recipients and providers) | 20% | Multiple time points | Ongoing, quarterly | Randomly |
| 4 | Bonevski 2016 | Fiore et al.’s System Changes approach [20, 28] and Ziedonis’ ATTOC model and Stages of Change | Unclear | Unclear | Unclear | Participant self-reported questionnaire | Participant self-reported questionnaire | Intervention and control group | Unclear | Unclear | Unclear | Unclear |
| 5 | Broekhuizen 2010 | Fidelity I-Change model of behaviour change, MI and MITI to assess fidelity and RE AIM framework for intervention fidelity | Unclear | Audiotaping | Audiotaping | Participant self-reported questionnaire | Participant self-reported questionnaire | Intervention group only (recipients and providers) | 100% | Before and During the intervention | Unclear | Unclear |
| 6 | Buhse 2013 | No | Unclear | In-person observation | Videotaping | Unclear | Participant self-reported questionnaire | Intervention group only (recipients and providers) | Unclear | Multiple time points | Unclear | Unclear |
| 7 | Busch 2015 | No | Unclear | Unclear | Interviews | Unclear | Unclear | Intervention group only (recipients and providers) | Unclear | At the end of the study only | Unclear | Unclear |
| 8 | Catley 2012 | MI | Unclear | In-person observation | Audiotaping In-person observation Supervision | Unclear | Unclear | Intervention group only (recipients and providers) | Unclear | Multiple time points | Unclear | Unclear |
| 9 | Croghan 2012 | No | Unclear | Roleplay | Audiotaping Supervision | In-person observation | In-person observation | Intervention group only (recipients and providers) | Unclear | During the intervention | Unclear | Randomly |
| 10 | Dahne 2018 | 5 A’S of smoking cessation | Unclear | Unclear | Participant self-reported questionnaire | Participant self-reported questionnaire |  | Intervention and control group | Unclear | Unclear | Unclear | Unclear |
| 11 | Duffy 2015 KPCHR | No - Based on interventions shown to be effective in Cochrane review | Unclear | Unclear | Provider self-report (checklist), Supervision | Data on p. use of online programme | Data on p. use of online programme | Intervention group only (recipients and providers) | Unclear | During the intervention | Unclear | Unclear |
| 12 | Duffy 2015 KU | No - Based on interventions shown to be effective in Cochrane review | Unclear | Provider self-report (checklist) In-person observation | In person observation, checklist | Collected Data On Calls | Provider self-report (checklist) | Intervention and Control group (recipients and providers) | Intervention 57% Control 51% | At the end of the study only | Unclear | Unclear |
| 13 | Duffy 2015 MGH | No - Based on interventions shown to be effective in Cochrane review | Unclear | Unclear | Audiotaping, Online Programme Data Collected | Online Programme Data Collected | Unclear | Intervention group only (recipients and providers) | Unclear | Unclear | Unclear | Unclear |
| 14 | Duffy 2015 NYU | No - Based on interventions shown to be effective in Cochrane review | Unclear | Roleplay | Audiotaping Provider self-report checklist | Participant self-reported questionnaire | Unclear | Intervention group only (recipients and providers) | Unclear | Multiple time points | Unclear | Randomly |
| 15 | Duffy 2015 UAB | No - Based on interventions shown to be effective in Cochrane review | Unclear | Unclear | Website Tracked Usage Data | Website Tracked Usage Data | Unclear | Intervention group only (recipients and providers) | Unclear | Unclear | Unclear | Unclear |
| 16 | Duffy 2015 UMMC | No - Based on interventions shown to be effective in Cochrane review | Unclear | In-person observation | Provider self-report (checklist) Participant self-reported questionnaire | Participant self-reported questionnaire | Participant self-reported questionnaire | Intervention and Control group (recipients and providers) | 11% (140 Nurses) | Multiple time points | Unclear | Unclear |
| 17 | Duffy 2015 USCD | No - Based on interventions shown to be effective in Cochrane review | Unclear | In-person observation Biweekly meetings to discuss delivery | Protocol adherence data collected | Participant self-reported questionnaire | Participant self-reported questionnaire | Intervention and Control group (recipients and providers) | Unclear | Multiple time points | Unclear | Unclear |
| 18 | El-Mohandes 2013 | Fidelity protocol implementation index (PII) | Unclear | Unclear | PII checklist used unclear who recorded data | Salivary cotinine levels and urine analysis | Unclear | Intervention group only (recipients and providers) | Unclear | At the end of the study only | Unclear | Unclear |
| 19 | Escoffery 2016 | social cognitive theory and stages of change | Unclear | Unclear | Unclear | Interviews | Interviews | Intervention group only (recipients and providers) | 192 of 227 p 85% | At the end of the study only | Unclear | Unclear |
| 20 | Gilbert 2017 | Taxonomy of behaviour change techniques | Unclear | Participant self-reported questionnaire | Audiotaping/compared to intervention protocol checklist | Participant self-reported questionnaire | Participant self-reported questionnaire | Control group only | Control: 41 taster sessions, 31.3% of all sessions delivered | At the end of the study only | Unclear | Randomly |
| 21 | Goenka 2010 | social cognitive theory, process assessment framework | Unclear | Workshops, manuals, role plays | Provider self-report (checklist) In person observation | Unclear | Provider self-report (checklist) In person observation | Intervention group only (recipients and providers) | Unclear | Multiple time points | Unclear | Unclear |
| 22 | Gould 2018 | Behaviour change wheel theoretical domains framework | Unclear | interviews | Participant self-reported questionnaire / interviews | Participant self-reported questionnaire | Participant self-reported questionnaire | Intervention and control group | Unclear | Multiple time points | Unclear | Unclear |
| 23 | Haas 2015 | Chronic Care Model and the Social Contextual Model for Reducing Tobacco Use | Unclear | Unclear | Unclear | Unclear | Interviews | Intervention group only (recipients and providers) | 100% | At the end of the study only | Once | All included |
| 24 | Halcomb 2015 | MI | Unclear | Interviews with staff | Interviews with staff | Unclear | Unclear | Intervention group only (recipients and providers) | 100% | At the end of the study only | Once | All included |
| 25 | Harter 2015 | Unclear | Unclear | Interviews | Interviews | Interviews | Participant self-reported questionnaire | Intervention group only (recipients and providers) | Unclear | At the end of the study only | Once | Unclear |
| 26 | Horn 2008 | MI | Unclear | In-person observation | Provider self-reported questionnaire | Participant self-reported questionnaire | Participant self-reported questionnaire | Intervention group only (recipients and providers) | Unclear | At the end of the study only | Unclear | Unclear |
| 27 | Johnson 2009 | No | Unclear | Unclear | Participant self-reported questionnaire | Participant self-reported questionnaire | Participant self-reported questionnaire | Intervention group only (recipients and providers) | Unclear | Multiple time points | 5 | Unclear |
| 28 | Kealey 2009 | Social cognitive theory with MI and CBT principles combined, Motivational Interviewing Treatment Integrity (MITI) Code | Audiotaping In person observation | In person observation | Audiotaping | Unclear | Unclear | Intervention group only (recipients and providers) | Unclear | Multiple time points | Once at end | Randomly |
| 29 | Leung 2017 | Unclear | Unclear | Unclear | Unclear | Participant self-reported questionnaire | Participant self-reported questionnaire | Unclear | Unclear | Unclear | Unclear | Unclear |
| 30 | Lycett 2010 | Behaviour change taxonomy, design, delivery, enactment, receipt | Unclear | Interview | Audiotaping Interview | Participant self-reported questionnaire, Co2 Measures | Participant self-reported questionnaire | Intervention group only (recipients and providers) | Unclear | Multiple time points | Unclear | Unclear |
| 31 | Matthews 2018 | Unclear | Unclear | Unclear | In person/observation | Participant self reported questionnaire | Participant self reported questionnaire | Intervention and control group | Unclear | At the end of the study only | Unclear | Unclear |
| 32 | McCambridge 2008 | Motivational interviewing, MITI for fidelity | Unclear | Unclear | Audiotaping | Participant self-reported questionnaire | Participant self-reported questionnaire | Intervention and control group (recipients and providers) | Unclear | Unclear | Unclear | Unclear |
| 33 | McClure 2017 | Social cognitive theory and cognitive behavioral therapy (CBT) also draws upon motivational interviewing and prospect theory | Unclear | Unclear | Audiotaping/Researcher checklist | Unclear | Participant self reported questionnaire | Intervention and control group | Intervention: 10%  Control: 30% | Unclear | Unclear | Randomly |
| 34 | Mujika 2014 | Motivational interviewing, MITI for fidelity | Unclear | Unclear | Audiotaping Provider self-report (checklist) | Unclear | Unclear | Intervention group only (recipients and providers) | 20% | During the intervention | Unclear | Randomly |
| 35 | Park 2006 | Motivational interviewing | Unclear | Unclear | Audiotaping, Supervision | Participant self-reported questionnaire Bogus Pipeline Procedure | Participant self-reported questionnaire | Intervention group only (recipients and providers) | 10% | Multiple time points | Ongoing | All included |
| 36 | Parker 2007 | Motivational interviewing - design | Unclear | Provider self-report | Audiotaping | Salivary cotinine levels | Unclear | Intervention group only (recipients and providers) | 10% | During the intervention | Unclear | Unclear |
| 37 | Pbert Fletcher 2006 | 5A model recommended by the US Public Health Service clinical practice guideline and the American Academy of Pediatrics. Design | Unclear | Study provider gave feedback, used checklist | Provider self-report | Participant self-reported questionnaire interviews & biochemical validation | Participant self-reported questionnaire interviews & biochemical validation | Intervention and control group (recipients and providers) | Unclear | During the intervention | Unclear | Unclear |
| 38 | Pbert, Osganian 2006 | Social cognitive theory, design  Stages of change theory, receipt | Unclear | Role play | Provider self-report (checklist)   Participant self-reported questionnaire | Participant self-reported questionnaire | Participant self-reported questionnaire | Intervention group only (recipients and providers) | 100% | Multiple time points | Unclear | All included |
| 39 | Richter 2016 | No | Unclear | Unclear | Audiotaping | Unclear | Unclear | Intervention group only (recipients and providers) | 10% | Unclear | Unclear | Unclear |
| 40 | Schlam 2018 | Unclear | Unclear | Unclear | Unclear | Participant self reported questionnaire | Participant self reported questionnaire | Control group only | Intervention: 100% | At the end of the study only | Unclear | All included |
| 41 | Schulz 2014 | I-Change model | Unclear | Unclear | Unclear | Length of time spent on website accessing online content | Participant self-reported questionnaire | Intervention group only (recipients and providers) | Unclear | Multiple time points | Unclear | Unclear |
| 42 | Sloboda 2009 | No | Unclear | Role play | Participant self-reported questionnaire In-person observation | Participant self-reported questionnaire | Participant self-reported questionnaire | Intervention group only (recipients and providers) | 20% of the lessons (two of 10 lessons) in seventh grade and 28% of the lessons (two of seven lessons) in ninth grade | Multiple time points | Each instructor was observed 4 times in each observation year. | All included |
| 43 | Spanou 2010 | Used MI principles and other psychology theories to create their own bcc framework | Unclear | Audiotaping, role play, consultations | Audiotaping | Participant self-reported questionnaire | Participant self-reported questionnaire | Intervention group only (recipients and providers) | Intervention N 15 participants, control N 15 participants, total N of each group unclear | Unclear | Unclear | Unclear |
| 44 | Taskila 2012 | No | Unclear | Role play | Audiotaping, interviews | Unclear | Unclear | Intervention group only (recipients and providers) | Approx 26 p | During the intervention | Unclear | Purposive |
| 45 | Taylor 2014 | Motivational interviewing and self-determination theory (SDT), social cognitive theory and control theory | Unclear | Unclear | Audiotaping, interviews with health trainers | Interviews with participants | Interviews with participants | Intervention and control group (recipients and providers) | 100% | Multiple time points | A sample of four participants for each of the three health trainers (12 participants in total) were selected to provide examples from early, late and in the middle of the study period | All included |
| 46 | Thyrian Freyer 2007 | Transtheoretical model (stages of change), design MI, MITI, delivery | Unclear | Unclear | Audiotaping, Supervision | Participant self-reported questionnaire | Participant self-reported questionnaire | Intervention group only (recipients and providers) | Unclear | Multiple time points | Unclear | Purposive |
| 47 | Thyrian Freyer 2010 | MI, MITI | Unclear | Unclear | Audiotaping, Supervision | Unclear | Unclear | Intervention group only (recipients and providers) | 54% of session were taped (161 of 299) and 84 taped sessions used (52.1%) | Multiple time points | Unclear | Unclear |
| 48 | Toll 2010 | No | Unclear | Audiotaping | Audiotaping, Supervision | Participant self-reported questionnaire | Participant self-reported questionnaire | Intervention group only (recipients and providers) | Unclear | During the intervention | Unclear | Unclear |
| 49 | Unrod 2016 | Stages of change model | Unclear | Unclear | Unclear | Participant self reported questionnaire | Participant self reported questionnaire | Intervention and control group | Intervention: 100% Control: 100% | At the end of the study only | Once | All included |
| 50 | Varvel 2010 | MI, design | Unclear | Unclear | Provider self-report | Participant self-reported questionnaire | Participant self-reported questionnaire | Intervention group only (recipients and providers) | 100% | At the end of the study only | Unclear | All included |
| 51 | Wang 2017 | AWARD model (ask about smoking history; warn about the high risk of smoking with the use of a health-warning leaflet; advise to quit as soon as possible and comply with the decided quit date; refer smokers to SC services; and do it again) | Unclear | Unclear | Unclear | Unclear | Biochemical Validation | Intervention and control group | Intervention: 100% Control: 100% | At the end of the study only | Once | All included |
| 52 | Wang 2018 | The advice was given using the structured model AWARD(ask about smoking history; warn about the high risk of smoking with the use of a health-warning leaflet; advise to quit as soon as possible and comply with the decided quit date; refer smokers to SC services; and do it again) | Unclear | Unclear | Unclear | Unclear | Biochemical Validation | Intervention and control group | Intervention: 100% Control: 100% | At the end of the study only | Once | All included |
| 53 | Webb 2007 | Unclear | Unclear | Unclear | Unclear | Participant self-reported questionnaire | Participant self-reported questionnaire | Intervention and control group (recipients and providers) | 100% | Multiple time points | Unclear | All included |
| 54 | White 2017 | Unclear | Unclear | Interviews | In person observation | Participant self-reported questionnaire / Interviews | Participant self-reported questionnaire | Intervention group only | Intervention: 100%  Control: unclear | During the intervention | Unclear | All included |
| 55 | Windsor 2014 | Unclear | Unclear | In person observation, supervision | Provider self-report (checklist) | Provider self-report (checklist), Biological Measures | Provider self-report (checklist), Biological Measures | Intervention group only (recipients and providers) | Unclear | Multiple time points | Unclear | Unclear |

# Supplementary table 5 Approaches to fidelity analysis and associations with intervention outcomes

|  | **Study (author/year)** | **Reliability or validity assessed** | **Sub-group analyses (i.e. variation in fidelity according to different factors)** | **Association between fidelity and study outcomes examined** | **Fidelity findings assessed and reported? (√ = reported, x = not reported, N = not assessed)** | | | | |
| --- | --- | --- | --- | --- | --- | --- | --- | --- | --- |
|  |  |  |  |  | **Design** | **Training** | **Delivery** | **Receipt** | **Enactment** |
| 1 | Asfar 2018 | Unclear | Unclear | Unclear | N | X | X | X | X |
| 2 | Blaakman 2013 | Unclear | Unclear | Unclear | N | N | **√** | **√** | **√** |
| 3 | Bock 2014 | Unclear | Unclear | Unclear | N | N | N | N | N |
| 4 | Bonevski 2016 | Unclear | Unclear | Unclear | N | N | X | X | X |
| 5 | Broekhuizen 2010 | Unclear | Unclear | Unclear | N | **√** | **√** | **√** | **√** |
| 6 | Buhse 2013 | Unclear | Unclear | Unclear | X | X | X | X | X |
| 7 | Busch 2015 | Unclear | Unclear | Unclear | N | N | **√** | N | N |
| 8 | Catley 2012 | Unclear | Unclear | Unclear | N | N | **√** | N | N |
| 9 | Croghan 2012 | Unclear | Unclear | Unclear | N | **√** | **√** | **√** | N |
| 10 | Dahne 2018 | Unclear | Unclear | Unclear | N | N | X | **√** | **√** |
| 11 | Duffy 2015 KPCHR | Unclear | Unclear | Unclear | N | N | **√** | X | X |
| 12 | Duffy 2015 KU | Unclear | Unclear | Yes but methods unclear | N | X | **√** | **√** | **√** |
| 13 | Duffy 2015 MGH | Unclear | Unclear | Unclear | N | N | **√** | N | X |
| 14 | Duffy 2015 NYU | Unclear | Unclear | Unclear | N | X | **√** | **√** | **√** |
| 15 | Duffy 2015 UAB | Unclear | Unclear | Unclear | N | N | X | **√** | X |
| 16 | Duffy 2015 UMMC | Unclear | Unclear | Unclear | N | X | X | X | X |
| 17 | Duffy 2015 USCD | Unclear | Unclear | Unclear | N | X | **√** | **√** | X |
| 18 | El-Mohandes 2013 | Unclear | Unclear | Unclear | N | N | **√** | N | N |
| 19 | Escoffery 2016 | Unclear | Unclear | Yes looked at receptivity to materials compared to smoking status | N | N | N | **√** | X |
| 20 | Gilbert 2017 | Unclear | Unclear | Yes, correlations for associations between adherence to protocol-specified behaviours and the length of session, and between adherence and the main outcome measures of attendance at the SSS and 7-day point prevalent abstinence at the 6-month follow-up. | N | N | **√** | **√** | **√** |
| 21 | Goenka 2010 | Unclear | Unclear | Unclear | N | **√** | **√** | **√** | N |
| 22 | Gould 2018 | Unclear | Yes, P and different healthcare professionals interviewed separately with regards to feasibility of delivery of intervention | Unclear | N | **√** | **√** | **√** | **√** |
| 23 | Haas 2015 | Unclear | Unclear | Yes (Examined use of each intervention component (ie, speaking to the TTS, receiving NRT, request or use of a community referral) and whether use of a specific component of the intervention was associated with quitting) | N | N | N | **√** | N |
| 24 | Halcomb 2015 | Unclear | Unclear | Unclear | N | **√** | **√** | N | N |
| 25 | Harter 2015 | Unclear | Unclear | Unclear | N | **√** | **√** | N | N |
| 26 | Horn 2008 | Unclear | Unclear | Unclear | N | X | **√** | **√** | **√** |
| 27 | Johnson 2009 | Unclear | Unclear | Yes (correlation between attendance at activities and the receipt of intervention by students) | N | N | **√** | **√** | **√** |
| 28 | Kealey 2009 | Yes | Unclear | Unclear | N | N | **√** | N | N |
| 29 | Leung 2017 | Unclear | Unclear | Unclear | N | N | N | **√** | **√** |
| 30 | Lycett 2010 | Unclear | Unclear | Unclear | N | N | **√** | N | N |
| 31 | Matthews 2018 | Unclear | Unclear | Unclear | N | N | **√** | **√** | **√** |
| 32 | McCambridge 2008 | Yes, compared fidelity of delivery across the four individual practitioners | Yes, in two treatment conditions | Yes, looked at delivery fidelity per individual practitioner compared to number of p reducing / quitting smoking | N | N | **√** | **√** | **√** |
| 33 | McClure 2017 | Unclear | Unclear | Unclear | N | N | X | N | N |
| 34 | Mujika 2014 | Unclear | Unclear | Unclear | N | N | **√** | N | N |
| 35 | Park 2006 | Unclear | Unclear | Unclear | N | N | **√** | **√** | **√** |
| 36 | Parker 2007 | Unclear | Unclear | Yes, cotinine-confirmed smoking status compared to  number of phone calls received | N | N | **√** | N | **√** |
| 37 | Pbert Fletcher 2006 | Unclear | Unclear | Unclear | N | **√** | **√** | **√** | **√** |
| 38 | Pbert, Osganian 2006 | Unclear | Unclear | Unclear | N | **√** | **√** | **√** | **√** |
| 39 | Richter 2016 | Unclear | Unclear | Yes, conducted cost-effectiveness analysis (incremental cost-effectiveness ratio) to evaluate the added cost per additional (1) enrollee in quitline and (2) quitter for warm handoff versus fax referral. An incremental cost-effectiveness ratio demonstrates the additional cost needed to achieve a better outcome when an intervention is more expensive and more effective. | N | N | X | N | N |
| 40 | Schlam 2018 | Unclear | Yes, different conditions | Yes, logistic regression to examine the relations between medication use and self-reported 7-day point-prevalence abstinence at weeks 8, 26, and 52. | N | N | N | N | **√** |
| 41 | Schulz 2014 | Unclear | Unclear | Yes, assessed exposure to intervention (total number of visits and time spent on website) in relation to behavioural outcomes for smoking | N | N | N | **√** | **√** |
| 42 | Sloboda 2009 | Unclear | Unclear | Unclear | N | X | **√** | X | X |
| 43 | Spanou 2010 | Unclear | Unclear | Yes, intend to examine participation in the seminars and use of software supported learning. Will map the clinicians’  use of the system (how often they log in, which pages  they use) in relation to the primary outcome. | N | X | X | N | N |
| 44 | Taskila 2012 | Unclear | Unclear | Unclear | N | N | X | N | N |
| 45 | Taylor 2014 | Yes | Unclear | Unclear | N | N | **√** | **√** | **√** |
| 46 | Thyrian Freyer 2007 | Unclear | Unclear | Yes, assessed predictive value of clients' characteristics on the counselers' MI-adherence with participant characteristics | N | X | **√** | **√** | **√** |
| 47 | Thyrian Freyer 2010 | Yes | Unclear | Yes, relationship between adherence to MI and smoking status after six months analysed | N | N | **√** | N | N |
| 48 | Toll 2010 | Unclear | Unclear | Unclear | N | N | **√** | N | N |
| 49 | Unrod 2016 | Unclear | Unclear | Yes, compared receipt of intervention leaflets with abstinence figures | N | N | N | **√** | **√** |
| 50 | Varvel 2010 | Unclear | Unclear | Unclear | N | N | **√** | **√** | **√** |
| 51 | Wang 2017 | Unclear | Unclear | Unclear | N | N | N | **√** | **√** |
| 52 | Wang 2018 | Unclear | Unclear | Unclear | N | N | N | **√** | **√** |
| 53 | Webb 2007 | Unclear | Unclear | Unclear | N | N | N | **√** | **√** |
| 54 | White 2017 | Unclear | Unclear | Unclear | N | N | **√** | **√** | **√** |
| 55 | Windsor 2014 | Unclear | Unclear | Compared intervention conditions to study outcomes (smoke free homes, CO confirmed quit) | N | X | **√** | **√** | **√** |
|  |  |  |  | **Total n of studies (% of total studies)** | 1 (1.8%) | 8 (14.5%) | 35 (63.6%) | 31 (56.4%) | 27 (49.1%) |

# Supplementary table 6 Analysis of fidelity data

|  | **Study (author/year)** | **How was fidelity data analysed / synthesised?** | | | | |
| --- | --- | --- | --- | --- | --- | --- |
|  |  | **Design** | **Training** | **Delivery** | **Receipt** | **Enactment** |
| 1 | Asfar 2018 |  |  |  | Participant questionnaire at baseline and the three-month follow-up to assess whether key points were learned, including the techniques and information discussed in the intervention sessions. |  |
| 2 | Blaakman 2013 | NA | NA | 20% of tapes were reviewed for fidelity, assessed with MITI scores. | Questionnaire to assess whether p. understood effects of second hand smoke on family | Questionnaire to assess whether p. understood what changes they could make to reduce SHS effects and intention to quit |
| 3 | Bock 2014 |  |  | Not reported |  |  |
| 4 | Bonevski 2016 |  |  | Cost-effectiveness analysis undertaken from the perspective of healthcare providers and patients, comparing the cost and effect from the organisational change intervention to usual care. Costs will be compared against the study’s primary outcome measure: 7-day-point prevalence for smoking abstinence at 6-week follow-up. Analysis will compare relative costs and outcomes in the intervention and control centres and report the incremental cost-effectiveness ratio (ICER). |  |  |
| 5 | Broekhuizen 2010 |  | MITI scores for sessions | MITI scores for sessions delivered |  |  |
| 6 | Buhse 2013 | Unclear | Providers practise counselling under supervision of a research fellow and subsequent feedback. | Video taped sessions examined to assess adherence to the counselling protocol. deviations from curriculum (duration, material use, content, didactics) were documented | Questionnaire used to assess knowledge of heart attack prevention strategies | Unclear |
| 7 | Busch 2015 |  |  | Interviews to assess if components were delivered (rated succeeded, failed or neutral) |  |  |
| 8 | Catley 2012 |  |  | Adherence to protocols for MI etc |  |  |
| 9 | Croghan 2012 |  |  | Looked at percentage of sessions that were considered adherent to the training, interventionist must have successfully met 6 of 7 of the counseling skills criteria | Participants monitored for adherence with ratings on attendance, homework completion and participation in topic being discussed. |  |
| 10 | Dahne 2018 |  |  | Participants asked to report whether physician or other healthcare provider: 1) asked about smoking status, 2) advised quitting smoking, 3) asked about willingness or readiness to quit smoking, 4) discussed medications for quitting, 5) advised medication use to quit smoking, 6) provided medication to quit smoking, and 7) provided a referral to the state smoking cessation quitline.. Findings not reported |  |  |
| 11 | Duffy 2015 KPCHR |  |  | Checklist to assess percentage of patients receiving info they should have according to protocol | Counselor documented consultation topics discussed with patient, assisted referral acceptance and referrals, and discharge medication orders. | Utilisation of quit resources documented at 6-month follow-up and electronic medical records where available |
| 12 | Duffy 2015 KU |  | Unclear | Trainers observed counselors’ delivery and recorded how well it was delivered | Counselor documentation and self-report at follow-up assessing if p received quit line services or other tobacco treatment | Counseling adherence data was collected from the quitline documenting the number of calls p completed |
| 13 | Duffy 2015 MGH |  |  | Medication delivered and number of calls delivered |  | Unclear |
| 14 | Duffy 2015 NYU |  | Counselors had weekly supervision with clinical supervisor | Number and duration of counselling calls recorded, success in reaching participants, NRT orders and topics covered. Calls were reviewed and assessed for adherence to the protocol and counseling approach. | 2-month follow-up surveys assessed patient satisfaction with treatment | Counselor documentation and 2-month patient follow-up surveys |
| 15 | Duffy 2015 UAB |  |  | Website tracked participant registrations and log-ins. Subset of participants were surveyed | Website tracked messages sent to and from the counselor | The website tracked participants’ web-site log-ins, number of days website accessed, and number of web pages visited |
| 16 | Duffy 2015 UMMC |  | Trained trainers until they demonstrated fidelity | Unclear | Unclear | Unclear |
| 17 | Duffy 2015 USCD |  |  | Reports were generated to show protocol adherence data. Timing, length, and frequency of counseling calls was recorded | Monitoring quitline counseling database,  patch delivery, and self-report at follow-up regarding receipt and use of patches, quitline or other treatment | Unclear |
| 18 | El-Mohandes 2013 |  |  | Number of sessions delivered to participants and protocol implementation index score |  |  |
| 19 | Escoffery 2016 |  |  |  | Questionnaire used to assess receptivity to intervention materials compared to smoking status |  |
| 20 | Gilbert 2017 |  |  | All transcripts anonymised and coded by two researchers, with 25% additionally coded by a third. Average inter-rater reliability for coding was 86% (range 68–99%) across sessions. The fidelity of each taster session was expressed as the percentage of overall protocol-specified behaviours that were delivered; Correlations were computed to explore associations between adherence to protocol-specified behaviours and the length of session, and between adherence and the main outcome measures of attendance at the SSS and 7-day point prevalent abstinence at the 6-month follow-up. | Looked at attendance at taster and subsequent smoking cessation sessions and abstinence rates |  |
| 21 | Goenka 2010 |  | Training feedback form assessing teacher’s satisfaction with the  program and materials. | Checklist showing which components were delivered: activities, postcard and poster tracking sheets | Questionnaires assessing enjoyment/participation of Posters, Games, Worksheets, Discussion,  Wrap-up, absorption while playing the games;  Proportion of the students in each class  participating in the discussion. |  |
| 22 | Gould 2018 |  | Fidelity of training timing, was the training delivered when it should have been? Three out of 6 services were trained in the month scheduled. One service was one month late due to the holiday period; two were two months late with training. | Qualitative data was analysed using a framework analysis and coded under categories of TDF, and BCW in addition to a general inductive analysis to capture other emergent themes. Capability, according to the BCW, comprises physical and psychological components (such as physical and cognitive skills, knowledge and behaviour regulation); opportunity includes physical and social aspects; and motivation includes reflective (through cognitive processes and intentions), and automatic (via habits, emotions or reinforcement). |  |  |
| 23 | Haas 2015 |  |  |  | Frequency of use of each intervention component assessed |  |
| 24 | Halcomb 2015 |  | Interviews looking at usefulness of training in delivery | Interviews looking at experience of delivery |  |  |
| 25 | Harter 2015 |  | Interviews looking at usefulness of training in delivery | Interviews to assess which intervention components they delivered, barriers and facilitators to this |  |  |
| 26 | Horn 2008 |  |  | Providers used MTI  assessment form to self-rate delivery, this was compared to manual | Questionnaires assessing usage of and usefulness of intervention components in relation to views on quitting | Questionnaires assessing acceptability of intervention components and whether quitting goals set |
| 27 | Johnson 2009 |  |  | Providers were observed and completed checklist highlighting delivery of key components | Questionnaire assessing which intervention activities students had accessed | Questionnaire assessing prevalence of smoking in students in last 7 days and last 30 days, compared this data with control schools |
| 28 | Kealey 2009 |  |  | Clinical supervision provided by clinical psychologists. Selected audio recordings of counseling calls reviewed. Counts of MI-specifi c behaviors, the mean number of MI-relevant  behaviors per call, SD and range calculate. Summary scores calculate to assess treatment  fidelity by comparing to established benchmarks for MI quality |  |  |
| 29 | Leung 2017 |  |  |  | Amount of physical activity p did and health related quality of life data, risk of disease |  |
| 30 | Lycett 2010 |  |  | Audio tapes of consultations assessed for fidelity to protocol and record keeping  assessed and monitored against protocol every few months. Deviations are recorded, discussed and corrected. |  |  |
| 31 | Matthews 2018 |  |  | Cofacilitator completed an in-session fidelity rating form that counted whether each specific intervention element required by the study protocol was addressed. | Participants rated their satisfaction with their smoking cessation counselor using a 5-point Likert scale on competency, communication skills, and overall satisfaction. |  |
| 32 | McCambridge 2008 |  |  | Percentage MITI summary scores were compared with recommended standards | Used questionnaire to assess understanding of harms of smoking, calculated odds ratios for reducing / quitting smoking compared to the fidelity of intervention they received from practitioner | Used questionnaire to assess reduction / quit behaviour, calculated odds ratios for reducing / quitting smoking compared to the fidelity of intervention they received from practitioner |
| 33 | McClure 2017 |  |  | Deviations from the protocol are shared with AW supervisors, so that protocol drift can be addressed with individual counselors. Summary reports of treatment fidelity metrics are reviewed routinely by the study team. |  |  |
| 34 | Mujika 2014 |  |  | Summary scores and global assessment scores were evaluated against established benchmarks for MI quality |  |  |
| 35 | Park 2006 |  |  | Compared delivery according to MI principles but didn’t report findings | Compared number of counselling calls received compared to smoking related outcome (e.g. smoking status, number of quit attempts) | Compared number of counselling calls received compared to smoking related outcome (e.g. smoking status, number of quit attempts) |
| 36 | Parker 2007 |  |  | Coded audiotapes of calls for presence of MI techniques and had supervision with MI expert |  | Cotinine-confirmed smoking status compared to  number of phone calls received |
| 37 | Pbert Fletcher 2006 |  | Providers practised intervention with director until provider could complete the intervention protocol | Patient report of intervention received, as assessed with patient exit interview to assess whether specific intervention steps were used. Scored out of 10 points (the PEI index score). | Questionnaire to assess confidence in ability to stop smoking (smokers) or remain smoke-free (nonsmokers) | Biochemical validation, The modified Fagerstrom Tolerance Questionnaire, The Hooked on Nicotine Checklist (HONC) |
| 38 | Pbert, Osganian 2006 |  | Role play by nurses to attain certification on intervention procedures | Questionnaire for nurses to assess what they delivered, compared to extent  intervention was implemented according to the protocol | Questionnaire for students to self-report on content of sessions and number attended, compared this to intervention protocol | Questionnaire for students to self report abstinence and assessed in relation to number of sessions attended |
| 39 | Richter 2016 |  |  | Research staff assessed fidelity using intervention checklist, calculated percentage of steps conducted correctly within each study arm, and reported performance back to quit line. |  |  |
| 40 | Schlam 2018 |  |  |  |  | Logistic regression used to examine the relations between medication use and self-reported 7-day point-prevalence abstinence at weeks 8, 26, and 52. |
| 41 | Schulz 2014 |  |  |  | Questionnaire ratings on satisfaction with intervention. Length of time spent on website assessed in relation to lifestyle risk factors score | Assessed exposure to intervention (total number of visits and time spent on website) in relation to behavioural outcomes for smoking |
| 42 | Sloboda 2009 |  | Role play. At the end of training, officers were asked to complete an anonymous training assessment form which measured the extent to which they were ready to teach the curriculum. | Content items present in intervention were added together and divided by the total number of activities to be covered, producing the proportion of activities covered for each lesson |  |  |
| 43 | Spanou 2010 |  | Feedback given on recorded consultations | Feedback given on recorded consultations |  |  |
| 44 | Taskila 2012 |  |  | Record some consultations and analyse content against schedule of proposed content |  |  |
| 45 | Taylor 2014 |  |  | Compared content delivered to manual, mean scores | Acceptability and feasibility of intervention to participants | Acceptability and feasibility of intervention to participants |
| 46 | Thyrian Freyer 2007 |  |  | Weekly group supervisions  held to maintain and ensure adherence to study protocol | Follow up calls with p to assess smoking status | Follow up calls with p to assess smoking status |
| 47 | Thyrian Freyer 2010 |  |  | Providers had supervision once or fortnightly, scored the sessions for adherence to MI principles using MITI |  |  |
| 48 | Toll 2010 |  |  | Weekly supervisions intended to help ensure a high level of adherence to the  protocol, including written and verbal feedback |  |  |
| 49 | Unrod 2016 |  |  |  | Participants in both of the Forever Free treatment groups were asked whether they received and read the intervention materials |  |
| 50 | Varvel 2010 |  |  | Questionnaire completed by lay health advisors to find out their experience of taking part. | Questionnaire completed by students to find out their experience of taking part | Questionnaire completed by students to find out their experience of taking part |
| 51 | Wang 2017 |  |  |  |  | Measured importance, difficulty and confidence in quitting and assessed it in relation to the quit rates for each group. Assessed cost effectiveness |
| 52 | Wang 2018 |  |  |  | Assessed rates of use of booklets and education cards. Participants levels of self perceived efficacy in quitting smoking were assessed. | Measured importance, difficulty and confidence in quitting and assessed it in relation to the quit rates for each group |
| 53 | Webb 2007 |  |  |  | Measured p expectations of intervention delivery and satisfaction, assessed intervention conditions in relation to applicability to their life, confidence about future  cessation, whether it changed smoking opinion and effect on intentions to quit | Measured p expectations of intervention delivery and satisfaction, measured readiness to quit with regards to the different conditions of intervention |
| 54 | White 2017 |  |  | Observed the delivery of the sessions and compared it to protocol | Qual interviews to ascertain the feasibility and acceptability of the sessions to trainers, students, parents and school staff | Qual interviews to ascertain the feasibility and acceptability of the sessions to trainers, students, parents and school staff |
| 55 | Windsor 2014 |  |  | Rated delivery against intervention delivery document, percentage score | Questionnaire asking about smoking habits and how useful they found SCRIPT, compared intervention conditions to study outcomes (smoke free homes, CO confirmed quit) | CO and salivary cotinine measures for participants, compared intervention conditions to study outcomes (smoke free homes, CO confirmed quit) |
